# Cold temperature and aridity shape the evolution of drought tolerance traits in Tasmanian species of *Eucalyptus*

**DOI:** 10.1093/treephys/tpad065

**Published:** 2023-05-19

**Authors:** Gabrielle E Hartill, Chris J Blackman, Benjamin Halliwell, Rebecca C Jones, Barbara R Holland, Timothy J Brodribb

**Affiliations:** ARC Centre of Excellence for Plant Success in Nature and Agriculture, College of Sciences and Engineering, School of Natural Sciences, University of Tasmania, Churchill Avenue, Sandy Bay, TAS, Australia; ARC Centre of Excellence for Plant Success in Nature and Agriculture, College of Sciences and Engineering, School of Natural Sciences, University of Tasmania, Churchill Avenue, Sandy Bay, TAS, Australia; ARC Centre of Excellence for Plant Success in Nature and Agriculture, College of Sciences and Engineering, School of Natural Sciences, University of Tasmania, Churchill Avenue, Sandy Bay, TAS, Australia; ARC Centre of Excellence for Plant Success in Nature and Agriculture, College of Sciences and Engineering, School of Natural Sciences, University of Tasmania, Churchill Avenue, Sandy Bay, TAS, Australia; ARC Centre of Excellence for Plant Success in Nature and Agriculture, College of Sciences and Engineering, School of Natural Sciences, University of Tasmania, Churchill Avenue, Sandy Bay, TAS, Australia; ARC Centre of Excellence for Plant Success in Nature and Agriculture, College of Sciences and Engineering, School of Natural Sciences, University of Tasmania, Churchill Avenue, Sandy Bay, TAS, Australia

**Keywords:** conductance, freezing tolerance, water relations, xylem cavitation

## Abstract

Perennial plant species from water-limiting environments (including climates of extreme drought, heat and freezing temperatures) have evolved traits that allow them to tolerate these conditions. As such, traits that are associated with water stress may show evidence of adaptation to climate when compared among closely related species inhabiting contrasting climatic conditions. In this study, we tested whether key hydraulic traits linked to drought stress, including the vulnerability of leaves to embolism (P_**50 leaf**_) and the minimum diffusive conductance of shoots (*g*_**min**_), were associated with climatic characteristics of 14 Tasmanian eucalypt species from sites that vary in precipitation and temperature. Across species, greater cavitation resistance (more negative P_**50 leaf**_) was associated with increasing aridity and decreasing minimum temperature. By contrast, *g*_**min**_ showed strong associations with aridity only. Among these Tasmanian eucalypts, evidence suggests that trait variation is influenced by both cold and dry conditions, highlighting the need to consider both aspects when exploring adaptive trait–climate relationships.

## Introduction

Water is a vital resource necessary for all functions of plant life (McElrone et al. 2013, Letcher 2022). In environments where water is a limiting resource (i.e., most terrestrial environments), perennial plants require a means of tolerating water deficit while minimizing negative impacts on growth, survival and reproduction (Zweifel et al. 2016, Bourbia et al. 2020). Strategies for tolerating water deficit include limiting water usage, reducing water loss and minimizing tissue damage during drought (Bray 1997, De Micco and Aronne 2012). These are achieved primarily through adaptation of morphological or physiological traits, including changes in cuticular properties (minimizing water loss; Lanning et al. 2020) and cavitation resistance (minimizing damage; Urli et al. 2013).

Species inhabiting particularly dry or hot climates have evolved various traits associated with water conservation or stress tolerance strategies (Osakabe et al. 2014). As the primary sources of water loss from the plant, leaves are important sites of adaptation to drought. One adaptation to seasonal water shortage is the loss of leaves altogether (drought deciduousness); however, evergreen trees—which maintain a proportion of their canopy during drought or other stresses—have instead evolved other strategies to tolerate water deficit (Vargas et al. 2021). Under drought conditions, leaves close their stomata to reduce water loss, but low levels of transpiration persist due to the permeability of the cuticle and incomplete stomatal closure. This residual water loss is quantified as the minimum leaf (or shoot) conductance to water vapor, *g*_min_ (Duursma et al. 2019). For species adapted to arid environments, where dry and hot conditions associated with high vapor pressure deficit (VPD) cause rapid water loss, low *g*_min_ is advantageous in conserving water during drought and delaying the onset of dehydration injury to the plant, in particular by xylem embolism formation (Cochard 2021). Nevertheless, while *g*_min_ often remains relatively constant at lower temperatures, it can increase rapidly at higher temperatures (Slot et al. 2021) due to changes in the structure of the cuticle (Schuster et al. 2016). The temperature at which *g*_min_ begins to rapidly increase is called the phase transition temperature (*T*_P_). Early evidence suggests higher *T*_P_ may be adaptive in arid environments (Bueno et al. 2019). However, few studies to date have compared variation in *T*_P_ among groups of species from contrasting environments.

Another key limitation for plant function under water stress is xylem cavitation, which can cause extensive tissue damage and eventually death from water loss (Brodribb et al. 2021). During drought, increasing xylem tension associated with decreasing plant water potential causes xylem cavitation—the entry and rapid expansion of air bubbles within the xylem—which blocks water transport (Vilagrosa et al. 2012). In arid environments, plants tend to exhibit greater resistance to cavitation in stems (Brodribb and Hill 1999, Choat et al. 2012) and leaves (Blackman et al. 2012, Scoffoni and Sack 2017) than those in wetter environments. Cavitation vulnerability is typically quantified as the water potential at which 50% loss of xylem conductivity occurs (P_50_). Following cavitation events, impaired water transport in leaves can lead to leaf desiccation (Brodribb et al. 2021). Therefore, in climates with greater cavitation risk, plants tend to withstand lower water potentials before they cavitate (i.e., lower P_50_).

Freezing in the soil or the plant vascular system may also impose limits to water supply, raising the possibility that similar traits may evolve in response to dry and freezing conditions (Charrier et al. 2014). Under freezing conditions, xylem embolisms can occur via ice nucleation or ice dehydration (Fernández-Pérez et al. 2018). Ice nucleation leads to the formation of gas bubbles when water in the xylem freezes and thaws, resulting in embolism (Lintunen et al. 2013). Ice dehydration occurs due to the lower water potential of ice leading to cavitation events in a similar way to embolism formation during drought (Charra-Vaskou et al. 2015). Increased cavitation resistance may therefore also be an important trait for species inhabiting either arid or freezing climates (Mayr et al. 2006).

This study focuses on leaf traits associated with water loss and cavitation resistance in 14 Tasmanian *Eucalyptus* species (including 8 from subgenus *Symphyomyrtus* and 6 from subgenus *Eucalyptus*) that occupy a range of climatic conditions on the island. While leaf traits like cavitation vulnerability have been observed across multiple plant clades (Maherali et al. 2004, Brodribb et al. 2014, Larter et al. 2015, Skelton et al. 2018), the combination of traits linked to minimum conductance and xylem vulnerability has not been considered. As a diverse clade of trees with long-lived leaves and a distribution that includes extreme heat and freezing, eucalypts provide an ideal genus in which to study the evolution of climate–trait associations. This study focuses on three physiological leaf traits relevant to climatic tolerances: cavitation vulnerability (P_50 leaf_), minimum conductance (*g*_min_) and the phase transition temperature (*T*_P_). We hypothesized that greater cavitation resistance would be found in species from dry climates and in species from freezing climates. We also hypothesized that species from warmer and drier climates would have a lower minimum diffusive conductance to water vapor and higher phase transition temperature than those from cooler or wetter climates.

## Materials and methods

**Figure 1:**
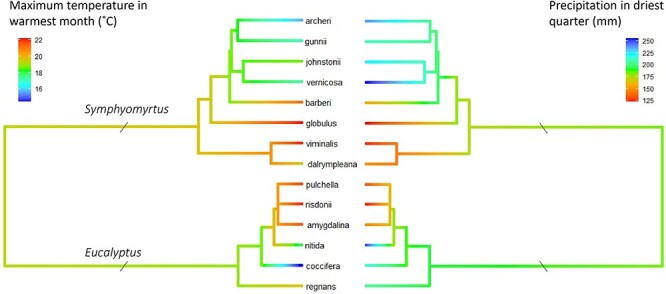
A grafted phylogenetic tree of Tasmanian *Eucalyptus* species, including eight species from subgenus *Symphyomyrtus* (Jones et al. 2016) and six species from subgenus *Eucalyptus* (Wooliver et al. 2017), with maximum temperature in the warmest month (°C; left), and precipitation in the driest quarter (mm; right) reconstructed via maximum likelihood and plotted as a color spectrum (colder/wetter climates in blue and warmer/drier climates in red). Maximum temperature in the warmest month ranged from 22 °C (*E. viminalis* and *E. risdonii*) to 15.8 °C (*E. archeri*). Precipitation in driest quarter ranged from 123 mm (*E. globulus*) to 256 mm (*E. vernicosa*).

**Figure 2:**
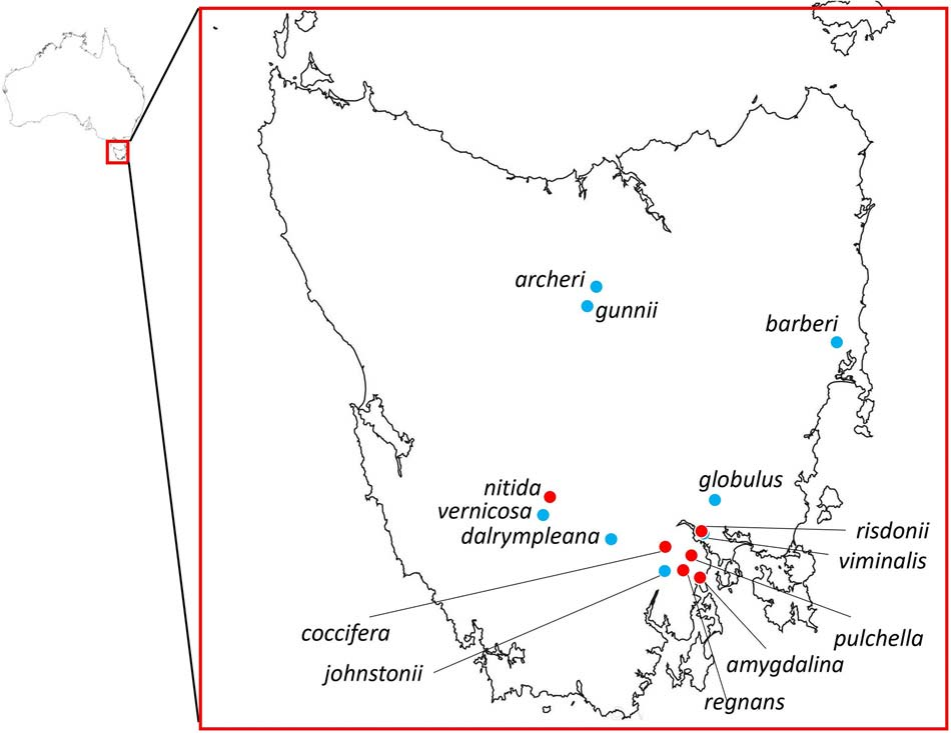
Sampling sites chosen around Tasmania for each of the fourteen *Eucalyptus* species, capturing a range of temperature and precipitation variation, as experienced by Tasmanian eucalypts (see Figure 1). Species in the *Symphyomyrtus* subgenus are represented in blue and species in the *Eucalyptus* subgenus are represented in red.

Plant material was collected from 14 climatically and phylogenetically diverse species of *Eucalyptus* subgenus *Symphyomyrtus* (Jones et al. 2016) and subgenus *Eucalyptus* (Wooliver et al. 2017) in Tasmania (Figure 1). Species and subsequent sampling sites were chosen to capture both high and low temperature and precipitation, as experienced by eucalypts in Tasmania (Table S1 available as Supplementary data at *Tree Physiology* Online; Figure 2). The Tasmanian climate is broadly cool temperate, with highly differentiated temperature and rainfall resulting from strong topographical and seasonal variation. Across the species sampling sites, mean annual temperature ranges from 6 °C in sub-alpine environments, where trees are subject to freezing temperatures in the winter months, to 12 °C in lower elevation and coastal environments, with mean maximum temperatures reaching 22 °C. Mean annual rainfall varies from 1500 mm in high-rainfall environments in western Tasmania to less than 600 mm in drier environments in the south-east. The climate variables considered when choosing species and representative populations were maximum temperature in the warmest month, minimum temperature in the coldest month, mean annual temperature, precipitation in the driest quarter and mean annual precipitation (all derived from BIOCLIM variables; Xu and Hutchinson 2011).

For each species, two branch samples of adult foliage (roughly 1 m each, from 4 to 6 m high on taller trees and ~1 m high on shorter species) were collected from each of five individual trees from each site. Sampling occurred in the morning or early afternoon during the cooler months of the austral autumn and winter of 2020, which helped ensure trees were well hydrated at the time of sampling. Branches were kept in plastic bags with wet paper towels to minimize dehydration during transport back to the laboratory.

### Cavitation vulnerability

To understand the vulnerability of the leaf xylem to cavitation during water stress, we allowed one branch from each of the five trees sampled per species to dry over multiple days while monitoring cavitation in leaf veins using the optical vulnerability technique (Brodribb et al. 2016). Plants were covered with plastic bags to prevent them from drying too rapidly. Psychrometers (Model PSY1, ICT International, Armidale, NSW, Australia) were attached to each branch, by removing the outer bark, clamping the sensor over the exposed vascular tissue and sealing the section off with parafilm. Water potential measurements were then taken automatically by the psychrometer every 10 min during branch dehydration. Water potential measurements were also manually taken every few hours, by placing one of the leaves into a Scholander pressure chamber (Model 1515D, PMS Instrument Company, Albany, Oregon, USA) and recording the balance pressure. One healthy fully expanded adult leaf from each branch (near the tip of the branch) was attached to a camera with an internal light source used to capture the formation of leaf vein embolisms (Cavicam, Hobart, TAS, Australia). The cavicam was positioned over a portion of the midrib and surrounding higher vein orders, with images taken every 3 min during the course of the bench dehydration.

Once the branch had completely dehydrated (i.e., when the leaves appeared brittle), image stacks were compared using the OSOV toolbox plugin in ImageJ (Version 1.53t)'s Fiji package. Images were subtracted from each other, and the image difference highlighted, with the differences occurring between images being established as cavitation events (see http://www.opensourceov.org/). Cavitation events seen in leaf veins were isolated from image noise due to leaf shrinkage. The cumulative area of cavitated pixels was then measured based on these changes occurring between images. This area was expressed as percentage of total cavitated area (at complete dehydration) and plotted against water potential (as measured by the psychrometer), creating a vulnerability curve (Figure S1 available as Supplementary data at *Tree Physiology* Online). From this vulnerability curve, the water potential at 50% accumulated embolism (P_50 leaf_) was determined per replicate, and a mean P_50 leaf_ calculated for each species.

### g_min_ and T_P_

We looked at two traits related to minimum shoot conductance during drought, to determine rates of residual water loss following stomatal closure (*g*_min_) and how this changed with increasing temperature (*T*_P_). Seven small branchlets (with several healthy leaves) were sampled from the second branch sampled from each of five trees per species (at the same time as branches were collected for cavitation vulnerability measurements). Branches were excised and paraffin wax used to seal the cut stems. Minimum shoot conductance was measured at different temperatures (25, 28, 32, 36, 40, 44 and 48 °C) inside a controlled temperature drying oven with a fan running to reduce boundary layer resistance. A single branchlet from each tree (*n* = 5) was used to measure *g*_min_ at each of the seven temperature steps, respectively. Changes in branchlet mass were recorded using a balance (Mettler Toledo, resolution = 0.1 mg, Model MS204S, Port Melbourne, VIC, Australia) every 10–20 min, whereby samples were briefly removed from the oven and weighed, until the rate of water loss from each branchlet reached a steady state following stomatal closure. At each temperature, relative humidity was measured, using a humidity sensor placed within the dehydrating oven. At the end of each dehydration experiment, leaves were separated from the stem and oven dried at 60 °C for 72 h for determination of total leaf dry mass. Total branchlet leaf area was calculated by multiplying total leaf dry mass by the species mean specific leaf area (SLA; m^2^ kg^−1^). Specfic leaf area was determined for each species from five representative leaves from each main branch (separate from those used to measure *g*_min_). Leaves were scanned and their area measured using ImageJ, before being dried down at 60 °C for 72 h for determination of dry mass.

Changes in branch mass were used to indicate the mass of water lost from the leaves between measurements. Using data associated with post-stomatal closure rates of water loss, transpiration rate (*R*) was calculated from changes in water loss (*w*) and time (*t*) and divided by the branchlet’s total leaf area (double-sided; A):


$$R=\Delta w/\left(\Delta t\times A\right).$$

From this slope, the leaf diffusive conductance (*g*_min_; mmol m^−2^ s^−1^) was calculated from the vapor-pressure deficit (VPD (kPa); calculated using the saturated vapor pressure (SVP (kPa); calculated using temperature (*T*)) and relative humidity (RH) (humidity and temperature measured using a humidity-temperature sensor) and the atmospheric pressure at sea level (101.6 kPa) according to the Arden Buck Equation (Buck 1981); see below):


$$\mathrm{SVP}=\left(610.7\times{10}^{\left(7.5\times T/\left(237.3+T\right)\right)}\right)/1000,$$

$$\mathrm{VPD}=\left(1\kern0.33em -\kern0.33em \left(\mathrm{RH}/100\right)\right)\times \mathrm{SVP},$$

$$g=R/\mathrm{VPD}\times 101.6.$$

The mean minimum leaf conductance to water vapor (*g*_min_; mmol m^−2^ s^−1^) was recorded for each species at each temperature (and *g*_min_ at a standard 25 °C used for comparison between species). Mean minimum conductance was then modeled against temperature, and the segmented R (Version 4.1.1) package used to detect a single breakpoint in the relationship, which we determined as the phase transition temperature (*T*_P_; °C; Figure S2 available as Supplementary data at *Tree Physiology* Online).

### Data analysis

**Figure 3:**
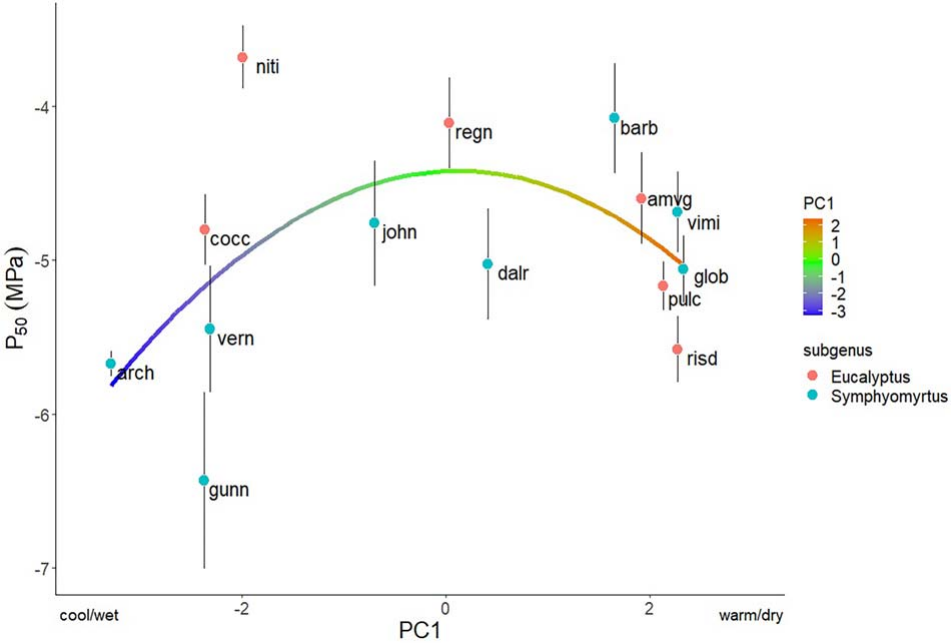
Loess smooth curve plotted over P_50 leaf_ and PC1, across all 14 species in both the *Symphyomyrtus* and *Eucalyptus* subgenus, with a high span to visualize the overarching trend of cavitation vulnerability across climate. Species are labeled according to their four-letter abbreviation (Table 1).

**Figure 4:**
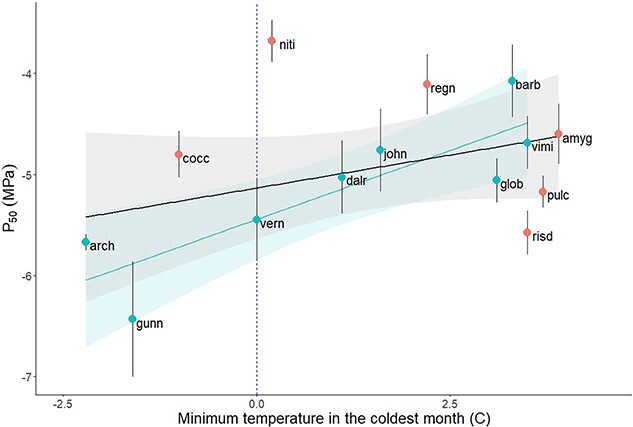
Phylogenetic generalized least squares (PGLS) regression (including confidence interval and standard error bars) for minimum temperature in the coldest month and P_50_. A regression line denoting the significant interaction of the *Symphyomyrtus* subgenus is plotted in blue (R^2^ = 0.42, *P* = 0.04). Species are labeled according to their four-letter abbreviation (Table 1). Subgenus is represented by point color (*Symphyomyrtus* in blue and *Eucalyptus* in red). Freezing point (0 °C) is indicated by the blue dotted line.

Five site-specific climate variables (mean annual temperature (°C; MAT), mean annual precipitation (mm; MAP), maximum temperature in the warmest month (°C), minimum temperature in the coldest month (°C) and precipitation in the driest quarter (mm)) were analyzed using a principal component analysis (PCA), yielding a principal component that explained 90.2% of variation (PC1; Figure S3 available as Supplementary data at *Tree Physiology* Online). Temperature and aridity were strongly correlated among sampling sites (Figure S4 available as Supplementary data at *Tree Physiology* Online; r = 0.72, *P* < 0.001), therefore PC1 was used for most subsequent analyses (analyses involving individual climate variables are included as supplementary material; Figure S5 available as Supplementary data at *Tree Physiology* Online). For each of the three traits measured (P_50 leaf_, *g*_min_ and *T*_P_) and PC1, we quantified the presence of phylogenetic signal using two separate methods; Blomberg’s K (Blomberg et al. 2003) and Pagel’s lambda (Pagel 1997, 1999), from a phylogenetic tree grafted from two separate trees (Jones et al. 2016, Wooliver et al. 2017) to include all species. We then performed phylogenetic generalized least squares (PGLS) regressions (using the caper R package) on each trait, using PC1 and subgenus as predictors (interaction terms non-significant in all cases and subsequently dropped) to account for the presence of phylogenetic covariance in model residuals. To help visualize the apparent parabolic relationship between PC1 and P_50 leaf_ across species, a Loess smooth curve with a high span (1.5) was fit to these data (Figure 3). Cook’s distance identified *Eucalyptus. gunnii* ssp. *divaricata* as an outlier in regressions involving *g*_min_. Models were subsequently explored with and without this outlier, and results excluding *E. gunnii* ssp. *divaricata* are presented. A regression was also performed between P_50 leaf_ and minimum temperature in the coldest month (which was strongly correlated with PC1; r = 0.96; Figure 4).

To control familywise error introduced by repeating P_50 leaf_ analyses on both climate variables (PC1 and minimum temperature in the coldest month), we applied a Bonferroni correction, after which all significant results remained significant. All analyses were performed in R Version 4.1.1 (R Core Team 2022).

## Results

**Figure 5:**
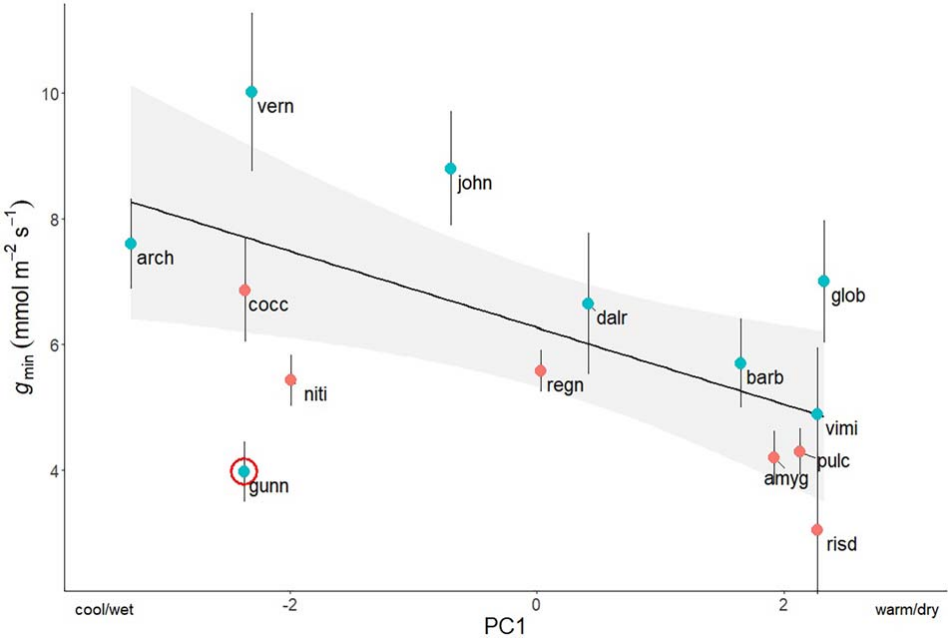
Phylogenetic generalized least squares (PGLS) regression for PC1 (with cooler/wetter climates to the left and warmer/drier climates to the right) and *g*_min_ (R^2^ = 0.34, *P* = 0.04), excluding a statistically significant outlier, *E. gunnii* ssp. *divaricata* (circled in red). Species are labeled according to their four-letter abbreviation (Table 1). Subgenus is represented by point color (*Symphyomyrtus* in blue and *Eucalyptus* in red).

Across all 14 Tasmanian species in this study, climatic niches at the site of sampling ranged from species experiencing freezing events (minimum temperatures below 0 °C, including *E. gunnii* ssp. *divaricata*, *E. archeri* and *E. coccifera*; Table 1) with relatively high levels of precipitation (>200 mm) in the driest quarter, to species experiencing maximum mean temperatures of > 22 °C in the warmest month (*E. globulus*, *E. risdonii* and *E. viminalis*; Table 1) and relatively low precipitation (<130 mm) in the driest quarter. Both cavitation resistance and cuticular permeability varied considerably between species across this climatic gradient (Figures 4 and 5).

**Table 1:** Climate and trait data for each eucalypt species, including maximum temperature in the warmest month, minimum temperature in the coldest month and precipitation in the driest quarter for each site from which the species was sampled, and mean values of P_50 leaf_*g*_min_, and *T*_P_ (including standard error in parentheses for P_50 leaf_ and *g*_min_; standard error not applicable for *T*_P_, as *T*_P_ values were extracted from a mean curve across all replicates of a species). Lower (more negative) P_50 leaf_ values equate to more cavitation-resistant xylem. Lower *g*_min_ equates to lower cuticular permeability (less water loss through cuticle), while higher *T*_P_ means lower conductance is maintained at higher temperatures.

Subgenus/species	Species abbreviation	Maximum temperature (°C)	Minimum temperature (°C)	Precipitation (mm)	P_50_ (MPa)	*g*_min_ (mmol m^−2^ s^−1^)	*T*_P_ (°C)
Subgenus *Symphyomyrtus*							
*E. archeri*	arch	15.8	−2.2	237	−5.67 (0.08)	7.60 (0.71)	35.83
*E. barberi*	barb	21.2	3.3	158	−4.08 (0.36)	5.70 (0.70)	34.17
*E. dalrympleana*	dalr	19.6	1.1	150	−5.03 (0.36)	6.64 (1.12)	40.86
*E. globulus*	glob	22.2	3.1	123	−5.06 (0.22)	7.00 (0.97)	37.38
*E. gunnii*	gunn	17.1	−1.6	212	−6.43 (0.57)	3.97 (0.48)	32.84
*E. johnstonii*	john	18.7	1.6	224	−4.76 (0.41)	8.80 (0.92)	34.60
*E. vernicosa*	vern	17.4	0	256	−5.45 (0.41)	10.01 (1.26)	29.78
*E. viminalis*	vimi	22	3.5	126	−4.69 (0.26)	4.88 (1.07)	35.24
Subgenus *Eucalyptus*							
*E. amygdalina*	amyg	21.4	3.9	148	−4.60 (0.30)	4.20 (0.42)	35.69
*E. coccifera*	cocc	14.5	−1	215	−4.80 (0.23)	6.86 (0.82)	31.70
*E. nitida*	niti	18	0.2	248	−3.68 (0.21)	5.43 (0.40)	31.59
*E. pulchella*	pulc	21.7	3.7	135	−5.17 (0.16)	4.29 (0.37)	35.74
*E. regnans*	regn	19.7	2.2	205	−4.11 (0.30)	5.58 (0.33)	35.89
*E. risdonii*	risd	22	3.5	126	−5.58 (0.21)	3.04 (1.02)	36.46

The species most vulnerable to cavitation, *E. nitida* (P_50 leaf_ =  −3.68 ± 0.46 MPa), was associated with high precipitation and mild temperatures, compared with other species (Table 1). Conversely, and despite experiencing high precipitation, the most cavitation-resistant species, *E. gunnii* ssp. *divaricata* (P_50 leaf_ = −6.43 ± 1.28 MPa), was exposed to some of the coldest temperatures. Across all species, P_50 leaf_ displayed a parabolic relationship with PC1, with more cavitation-resistant xylem (lower P_50 leaf_) in species inhabiting either cooler/wetter climates and (to a lesser extent) warmer/drier climates and less cavitation-resistant xylem in species occupying intermediate climates (Figure 3). When considering individual climate variables, significant interactions with subgenus emerged. Specifically, subgenus *Symphyomyrtus* showed a significant association between P_50 leaf_ and minimum temperature, driving the pattern of more resistant xylem in species from cooler climates (R^2^ = 0.42, *P* = 0.04). Conversely, only one species sampled from subgenus *Eucalyptus*, *E. coccifera*, was subject to freezing conditions, while the remaining species tended to show higher cavitation resistance in warmer climates (Figure 4).

Across all species, *g*_min_ decreased in warmer/drier climates (Figure 5; R^2^ = 0.34, *P* = 0.04), while *T*_P_ showed no significant correlation with climate (Figure S6 available as Supplementary data at *Tree Physiology* Online; R^2^ = −0.08, *P* = 0.62). The species with the highest *g*_min_, *E. vernicosa* (10.01 ± 2.82 mmol m^−2^ s^−1^), occurred in the highest precipitation site, while the species with the lowest *g*_min_, *E. risdonii* (3.04 ± 2.28 mmol m^−2^ s^−1^), were collected from a comparatively warm and dry site (Table 1). *Eucalyptus gunnii* ssp. *divaricata*, however, was a significant outlier in the relationships between PC1 and *g*_min_ (Cook’s D = 0.58), having a particularly low *g*_min_ relative to its climatic niche. Neither the three physiological traits nor the five climate variables showed evidence of phylogenetic signal in both K and lambda (*P* > 0.05; Table S2 available as Supplementary data at *Tree Physiology* Online).

## Discussion

This study shows that, in Tasmanian eucalypts, increased leaf xylem cavitation resistance is associated with species from both warmer/drier climates and cooler climates that are exposed to winter freezing. Despite previous studies indicating higher cavitation resistance is typically associated with species in warm/dry environments (Maherali et al. 2004, Brodribb et al. 2014, Larter et al. 2015, Skelton et al. 2018), we observed the strongest cavitation resistance among species inhabiting cool/wet environments. The most cavitation-resistant species in this study was the most cold-tolerant eucalypt, *E. gunnii* ssp. *divaricata* (Davidson and Reid 1985, Cauvin and Potts 1991), which exhibited a P_50 leaf_ equivalent to highly drought tolerant eucalypts from semi-arid regions (Blackman et al. 2014). Across all 14 species, we observed a clear parabolic relationship between P_50 leaf_ and PC1, suggesting that cavitation resistance has evolved in response to both frost (especially in subgenus *Symphyomyrtus*; though, across mainland Australia, *Symphyomyrtus* species tend to inhabit more arid climates; Hughes et al. 1996) and drought, in these Tasmanian eucalypts.

Though both drought and frost can cause xylem embolism, the process by which air bubbles form differs. The most cavitation-resistant species observed in this study occurred in environments where ice dehydration may drive the evolution of more negative P_50 leaf_ values in a manner similar to water deficit under drought (Charra-Vaskou et al. 2015). Eucalypts are not winter deciduous, therefore species occurring in freezing environments have instead adapted leaves that are highly resistant to embolism. The weak positive trend between cavitation resistance and aridity in Tasmanian eucalypts may be explained by the lack of especially hot-dry environments in Tasmania. This weak trend contrasts with strong links between increasing cavitation resistance and aridity observed among eucalypt species from mainland Australia, where some species occupy arid zone environments (Peters et al. 2021). In Tasmania, it appears that exposure to freezing has imposed stronger selection on cavitation resistance than aridity, with the lowest P_50 leaf_ values found among species occurring in the coldest climates.

In our sample of Tasmanian eucalypts, species from warmer and drier climates exhibited lower *g*_min_ than those in wetter and cooler climates. However, this relationship was only significant when *E. gunnii* ssp. *divaricata* (which had a significantly lower *g*_min_ than expected for its climate) was excluded. Generally, leaves from species inhabiting the driest climates (such as *E. viminalis* and *E. risdonii*) were better able to conserve water following stomatal closure during drought, while species from the wetter climates (*E. vernicosa* and *E. johnstonii*) lost water most rapidly. With water loss being particularly detrimental in drier climates, strong selection for low *g*_min_ under arid conditions is likely to be responsible. Due to higher evaporative demand associated with higher temperatures (Vicente-Serrano et al. 2022), such cuticle adaptations are also important in warmer environments. However, as temperature and precipitation were strongly correlated across the sampled sites (Figure S4 available as Supplementary data at *Tree Physiology* Online), it is unclear whether drought, heat or both have driven the adaptation of *g*_min_ in these climatic extremes.

*Eucalyptus gunnii* ssp. *divaricata* was a significant outlier, with one of the lowest *g*_min_ values among our sample group, despite occupying relatively cool and wet environments. Rather than an adaptation to heat/aridity, these low *g*_min_ values may represent properties of the leaves that help prevent damage from frost (Shepherd and Wynne Griffiths 2006); indeed, the subspecies of *E. gunnii* that we sampled is recognized as the most frost-tolerant eucalypt (Davidson and Reid 1985, Cauvin and Potts 1991). Nevertheless, despite its strong resistance to cavitation and low minimum conductance, recent reports of dieback in *E. gunnii* ssp. *divaricata* populations suggest the species is susceptible to high mortality rates under drought conditions (Potts et al. 2001, Calder and Kirkpatrick 2008). Further research is required, including a more detailed assessment of hydraulic properties and rooting depth, to better understand the drivers of these mortality events in *E. gunnii* ssp. *divaricata*.

Unexpectedly, no significant association was found between phase transition temperature and climate, suggesting that Tasmanian eucalypts have not evolved increased resistance to catastrophic water loss, where they inhabit warmer or drier climates. As *T*_P_ regressions are derived from mean *g*_min_ at each temperature (i.e., each point having uncertainty), the uneven variation within species meant that some *T*_P_ points were not easily distinguished or were not contextually sensible. Therefore, despite evidence for adaptation in *g*_min_, at a standard temperature, *T*_P_ in Tasmanian *Eucalyptus* species was not significantly related to either temperature or aridity.

## Conclusions

These results suggest that water availability and temperature (especially freezing) are both important drivers in the evolution of leaf hydraulic traits in Tasmanian eucalypts. Heat/drought appears to be a major driver in the evolution of *g*_min_, with species typically having a lower *g*_min_ in warm and dry climates. However, in Tasmania, tolerance to freezing appears to be a strong influence on the evolution of xylem cavitation in leaves. Sampling more species across a wider range of climates (particularly the arid regions of mainland Australia, and in climates that are either warm and wet or cool and dry) will provide further insight into the roles of temperature and moisture availability in the evolution of hydraulic traits in this ecologically and economically important genus.

## Supplementary Material

Supplementary_Figures_tpad065

Supplementary_Tables_tpad065

## Data Availability

The data that support the findings of this study are available from the corresponding author upon reasonable request.
