# Supplementary material for: Cold temperature and aridity shape the evolution of drought tolerance traits in Tasmanian species of Eucalyptus
Source: Tree Physiol. 2023 May 19;43(9):1493–500. doi: 10.1093/treephys/tpad065 (PMC10493950; doi:10.1093/treephys/tpad065)
Supplement: Supplementary_Figures_tpad065 [file supplementary_figures_tpad065.docx]

Supplementary Material

1. *E. archeri*


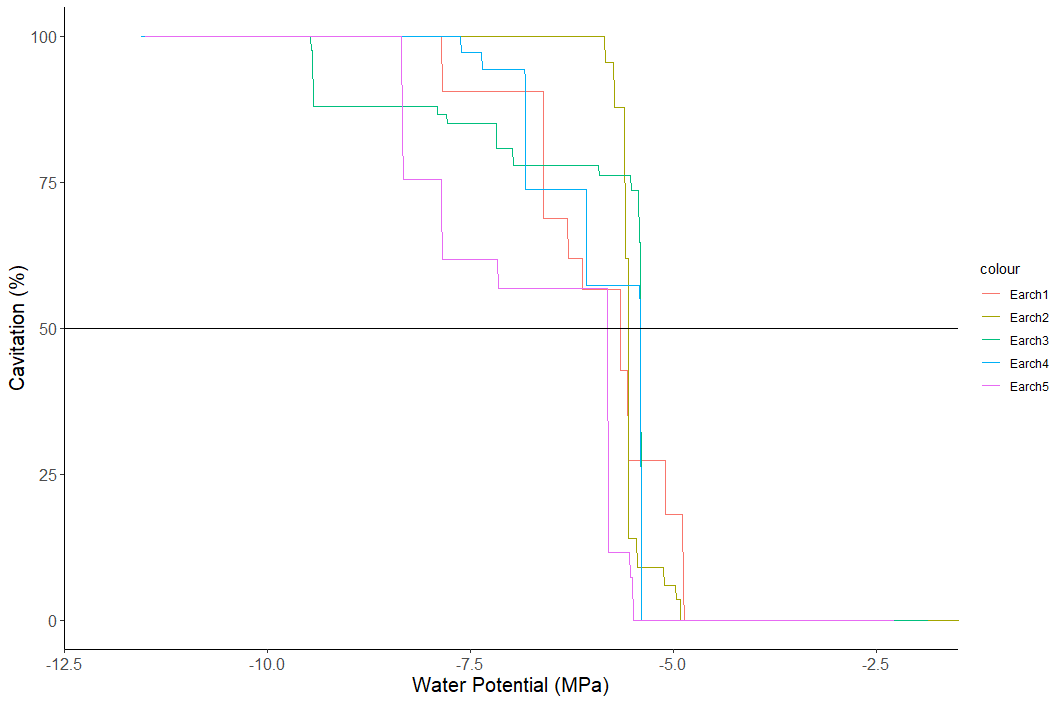


1. *E. barberi*


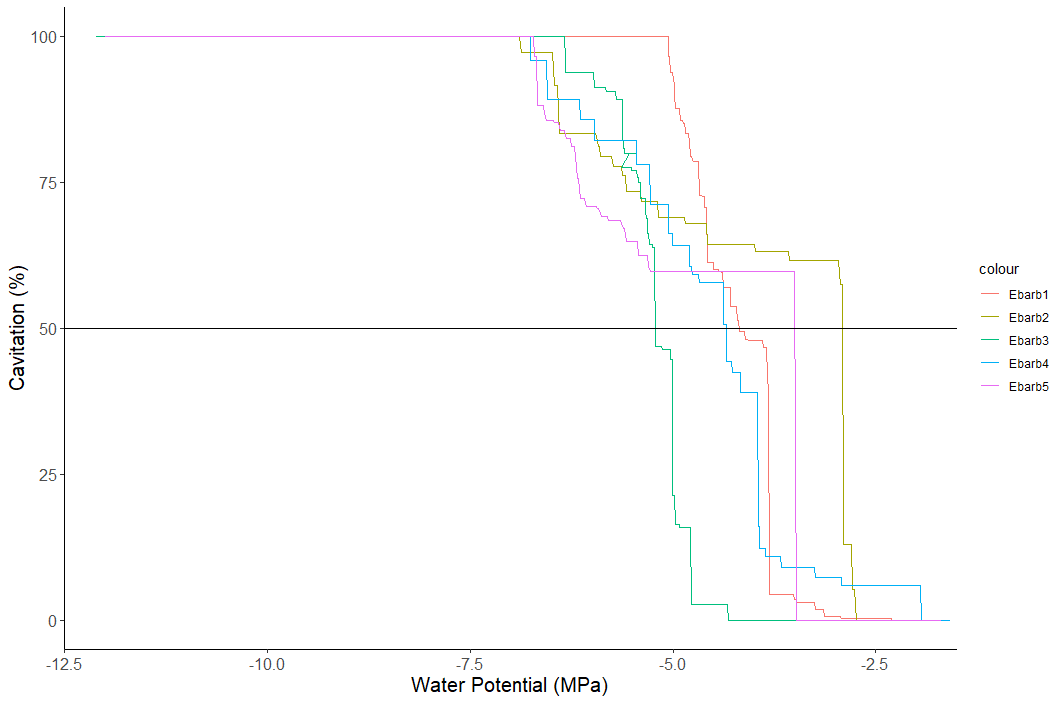


1. *E. globulus*


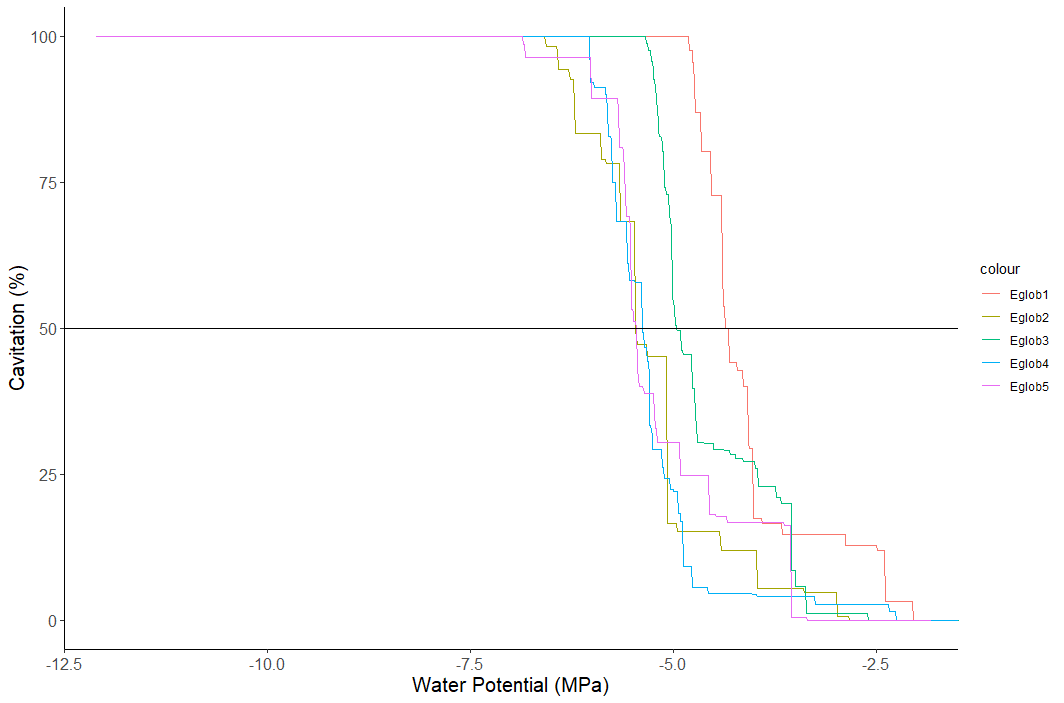


1. *E. gunnii*


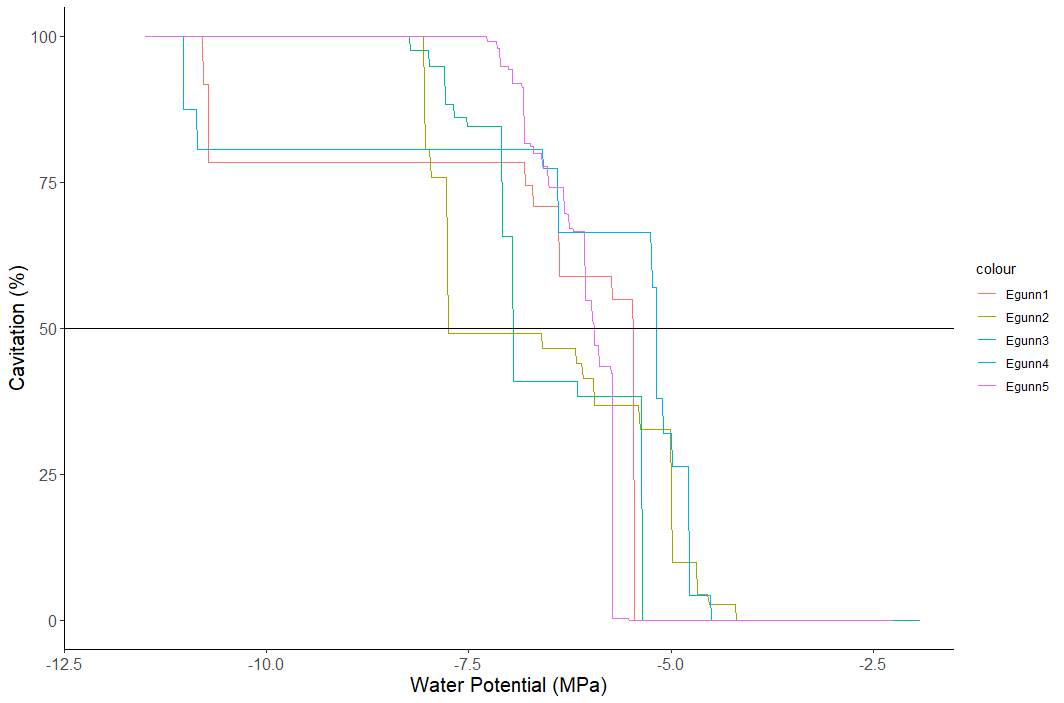


1. *E. johnstonii*


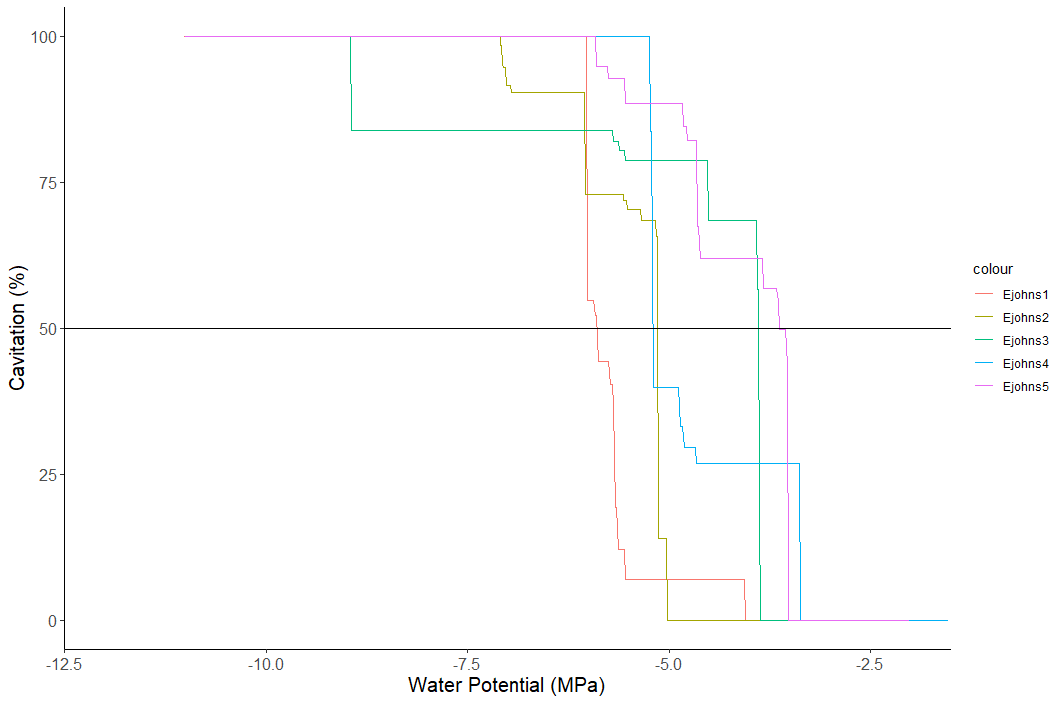


1. *E. viminalis*


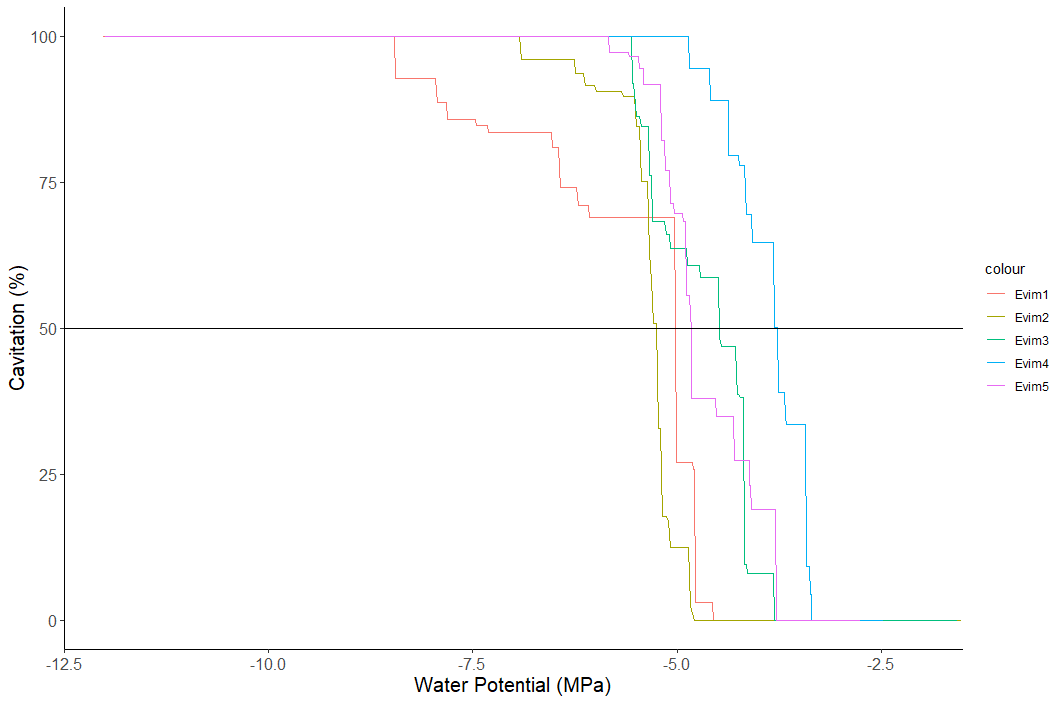


1. *E. dalrympleana*


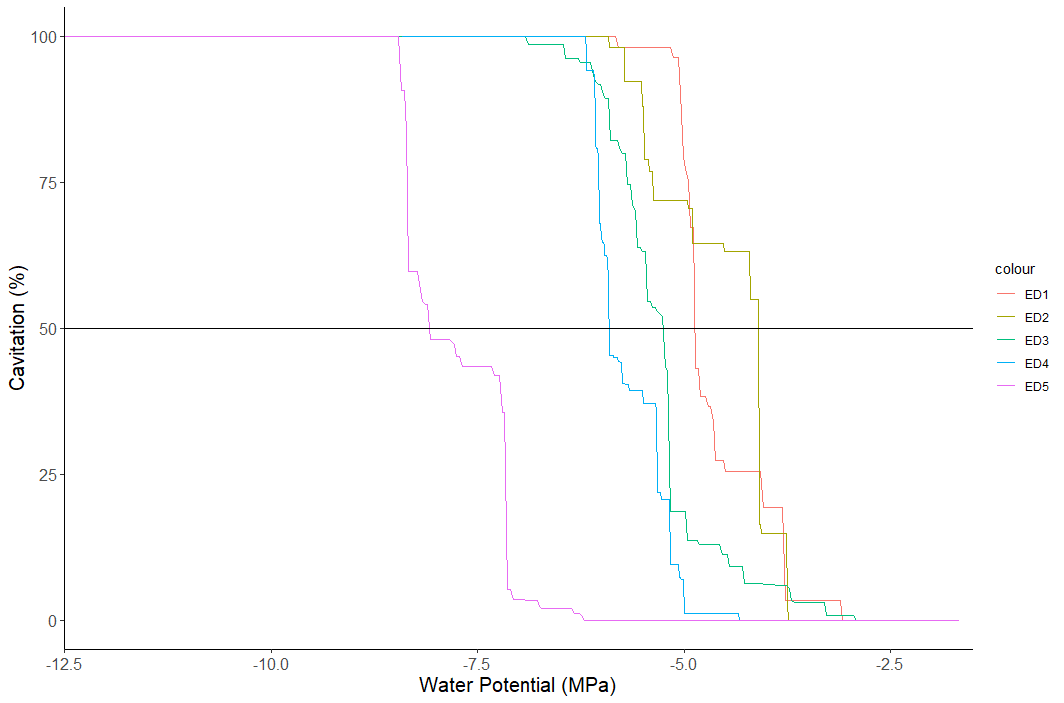


1. *E. vernicosa*


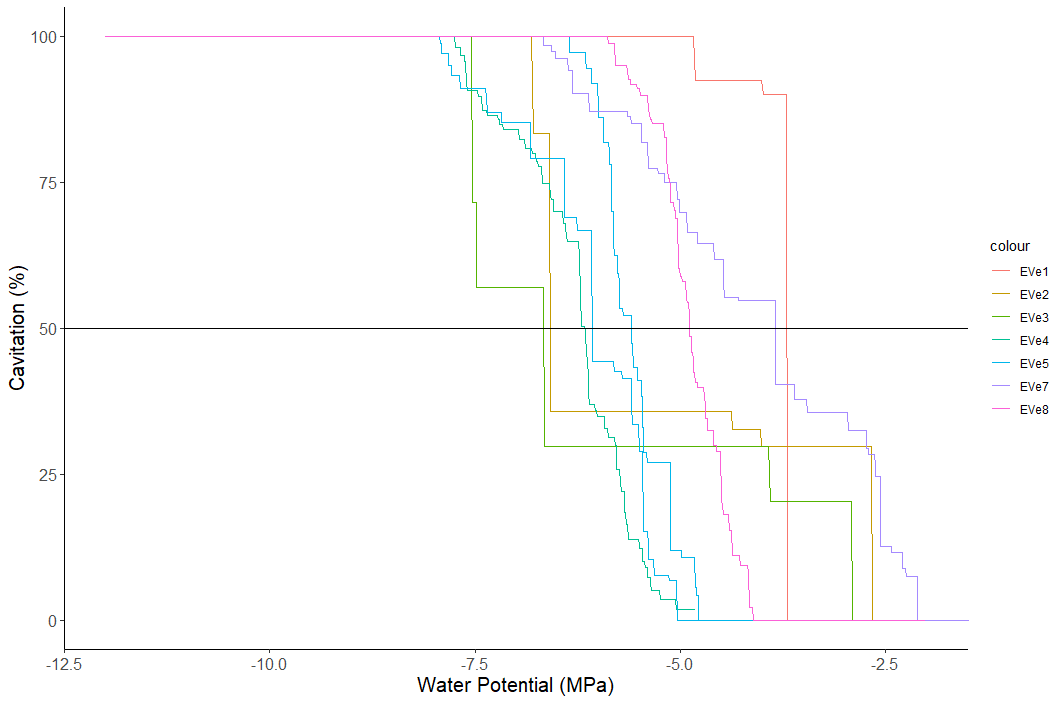


1. *E. amygdalina*


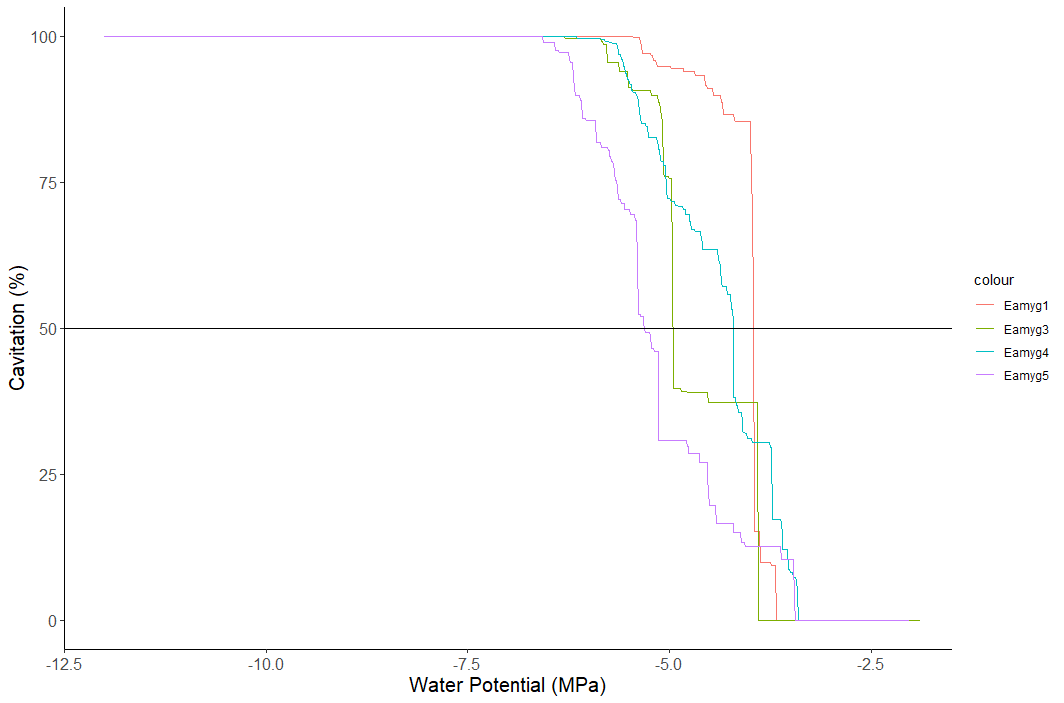


1. *E. coccifera*


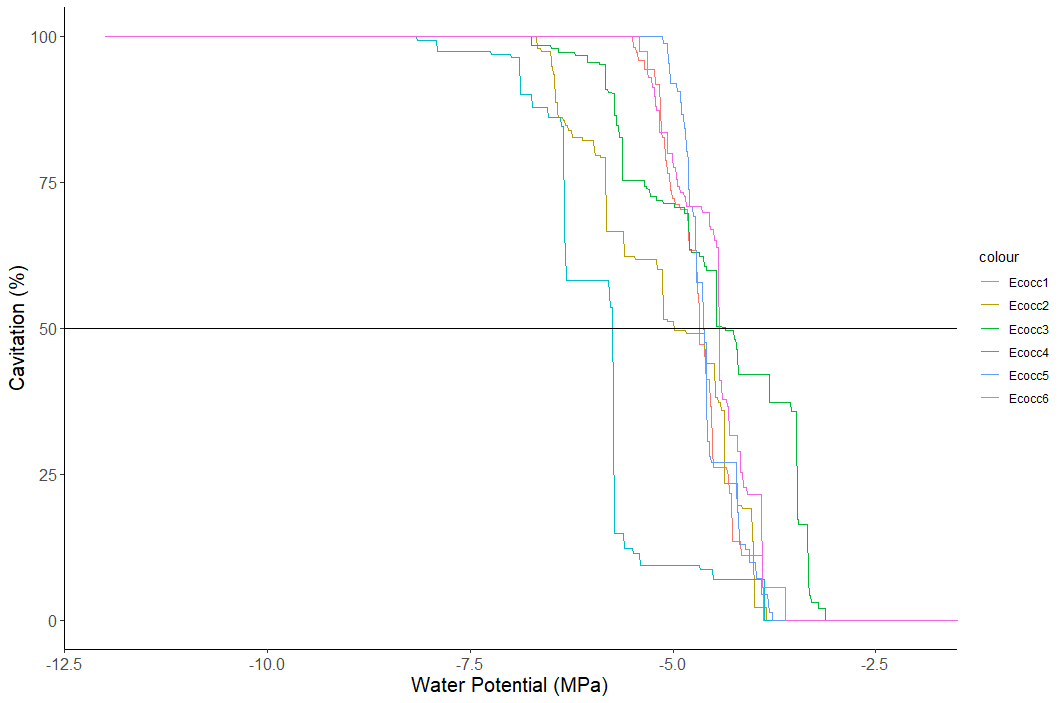


1. *E. nitida*


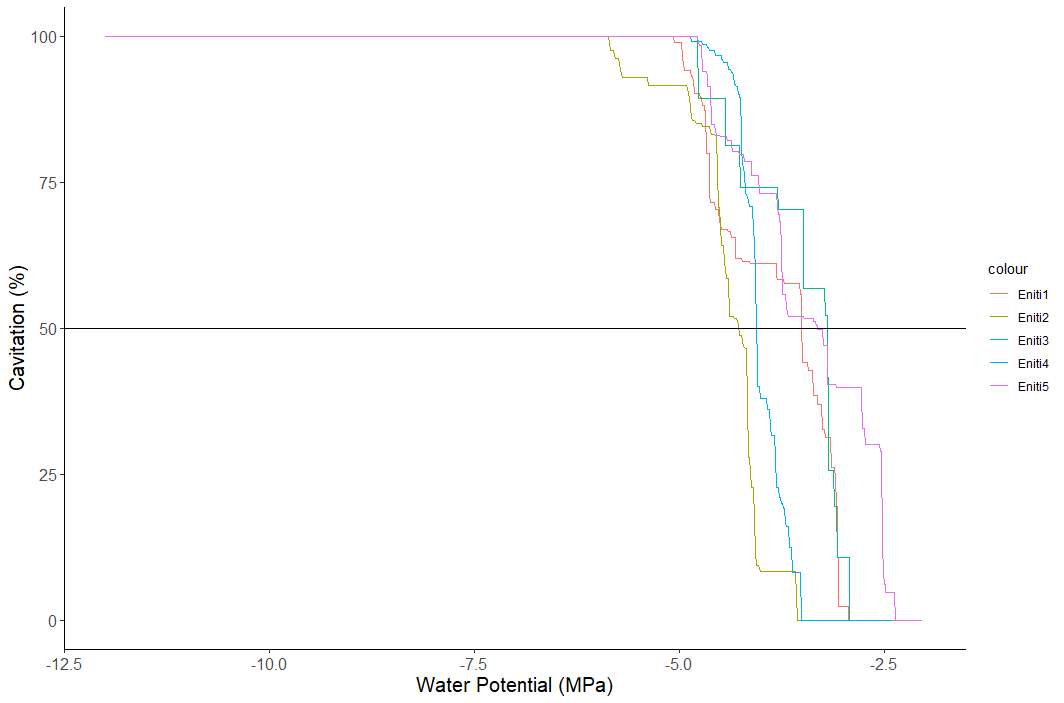


1. *E. pulchella*


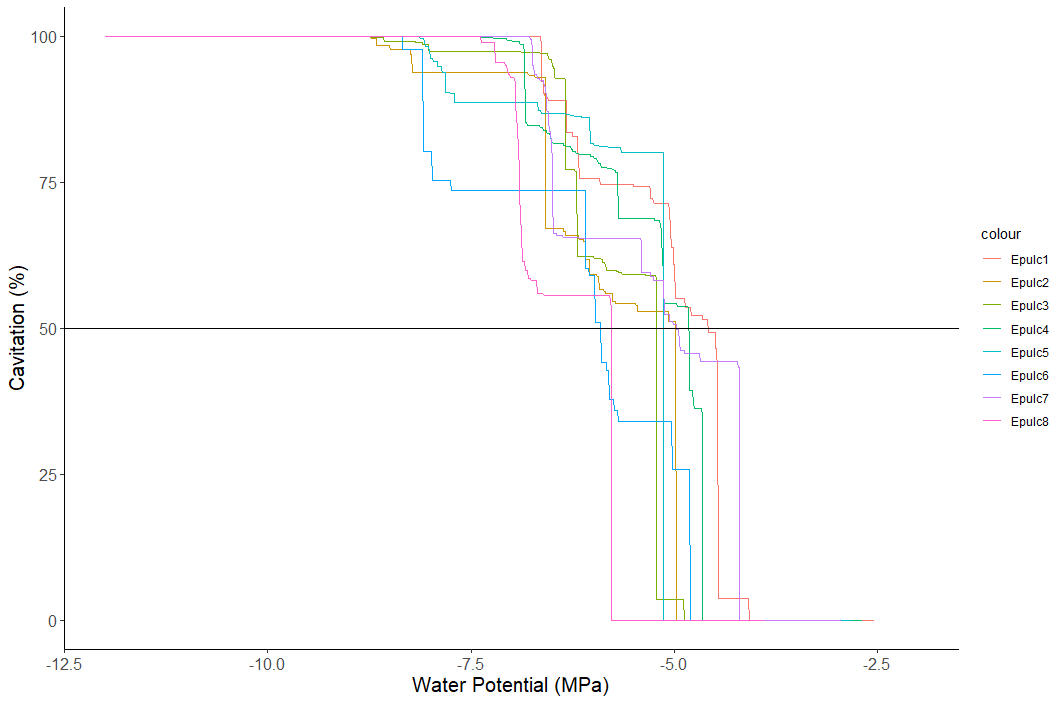


1. *E. regnans*


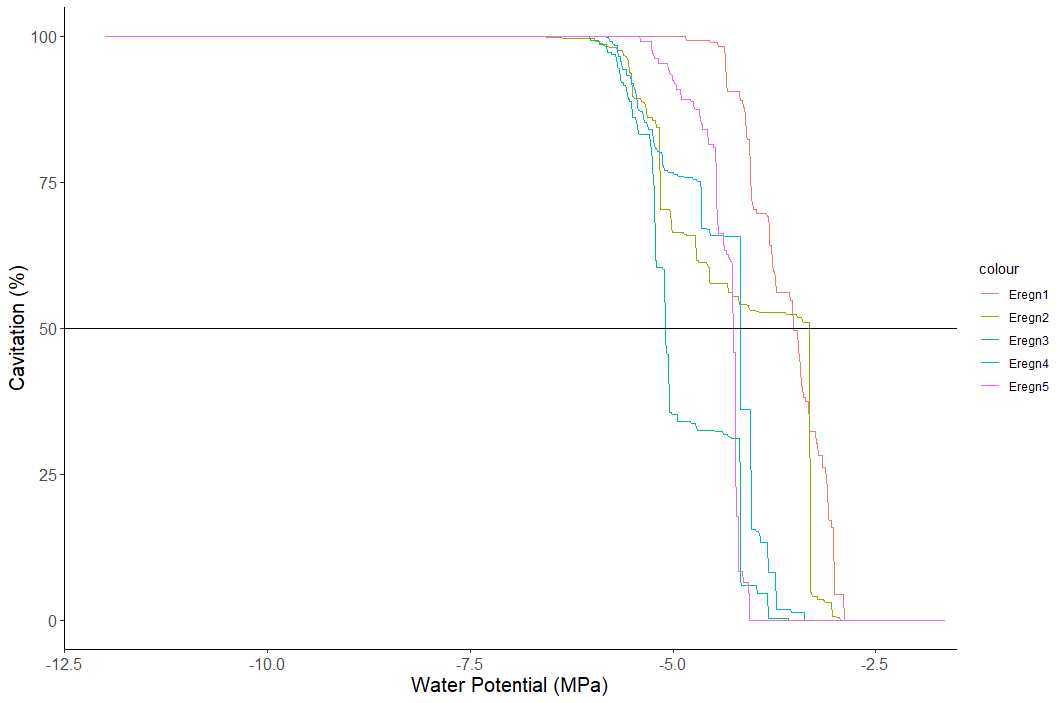


1. *E. risdonii*


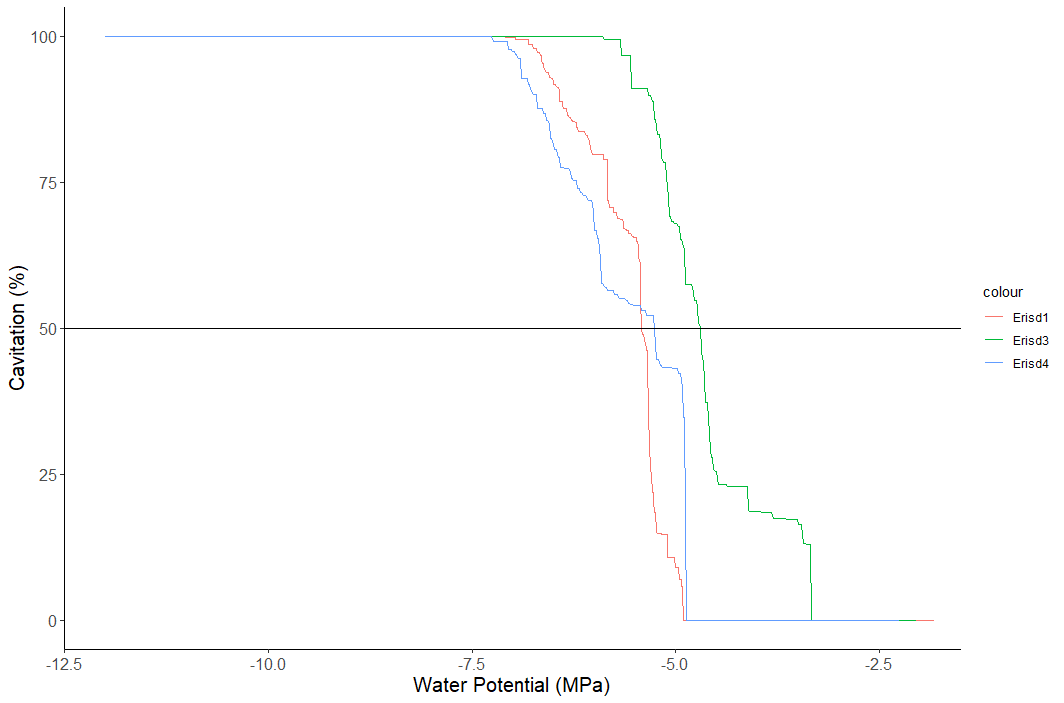


**Figure S1:** Vulnerability curves (percentage of cavitation plotted against water potential) for each of the five samples of fourteen Tasmanian *Eucalyptus* species: a) *E. archeri*, b) *E. barberi*, c) *E. globulus*, d) *E. gunnii*, e) *E. johnstonii*, f)  *E. viminalis*, g) *E. dalrympleana*, h) *E. vernicosa*, i) *E. amygdalina*, j) *E. coccifera*, k) *E. nitida*, l) *E. pulchella*, m) *E. regnans*, and n) *E. risdonii*. P_50_ is represented as the line at 50% cavitation, ranging from a mean of -6.43 ± 1.28 MPa (*E. gunnii*) to -3.68 ± 0.46 MPa (*E. nitida*).

1. *E. archeri*


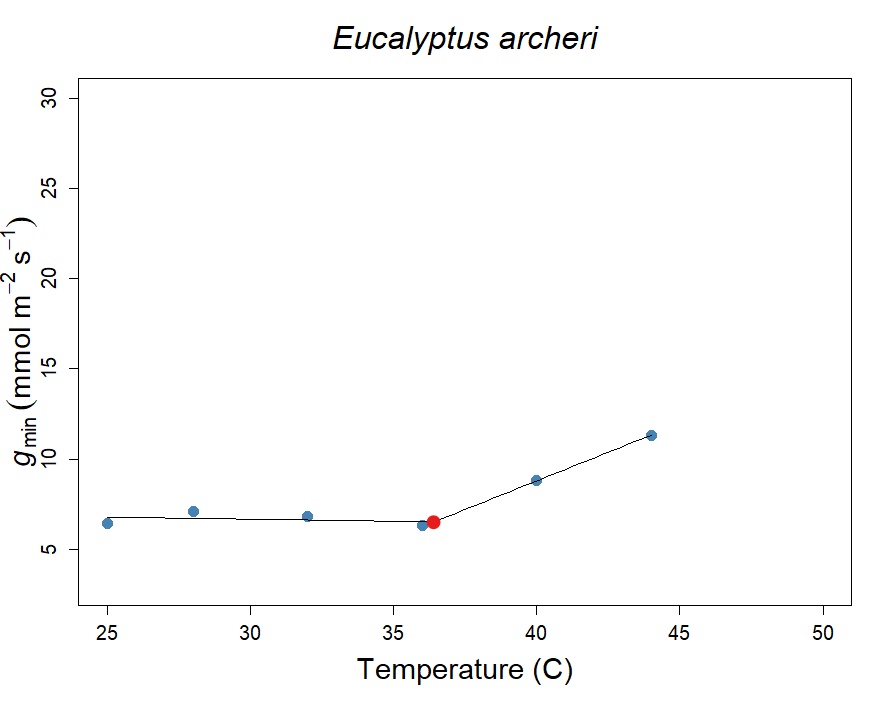


1. *E. barberi*


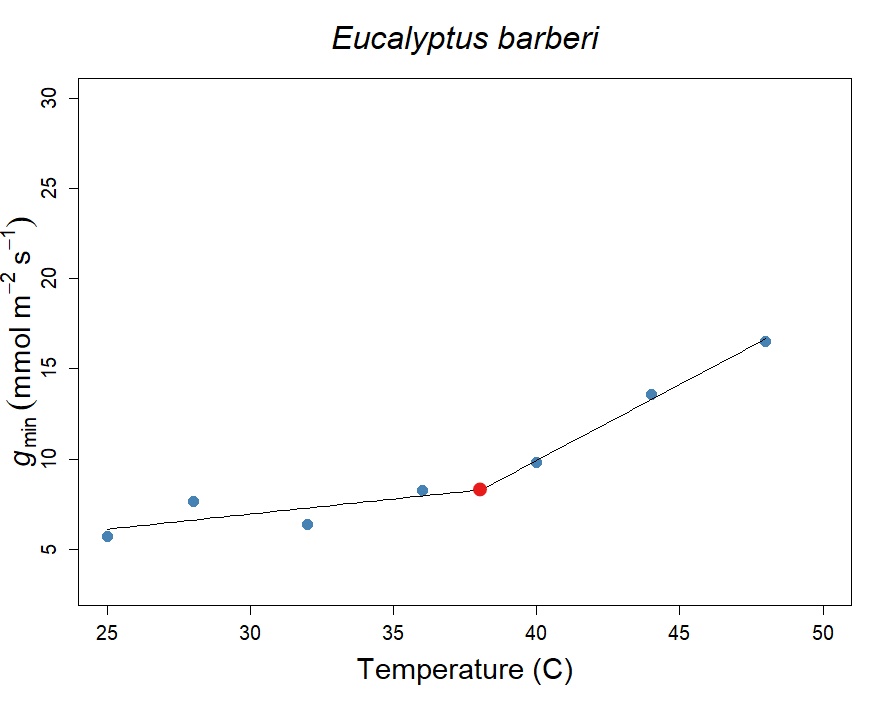


1. *E. globulus*


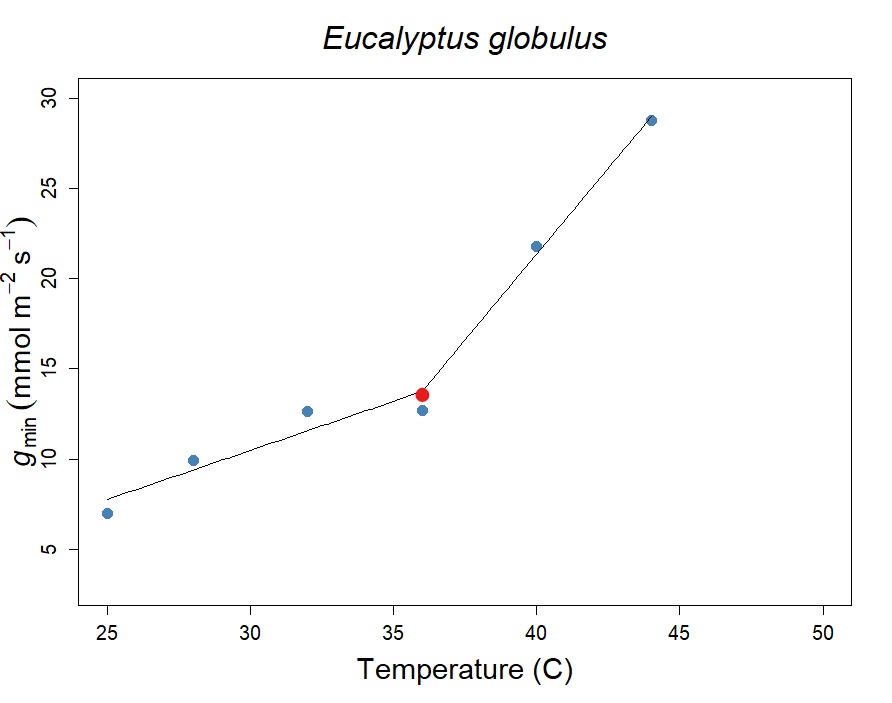


1. *E. gunnii*


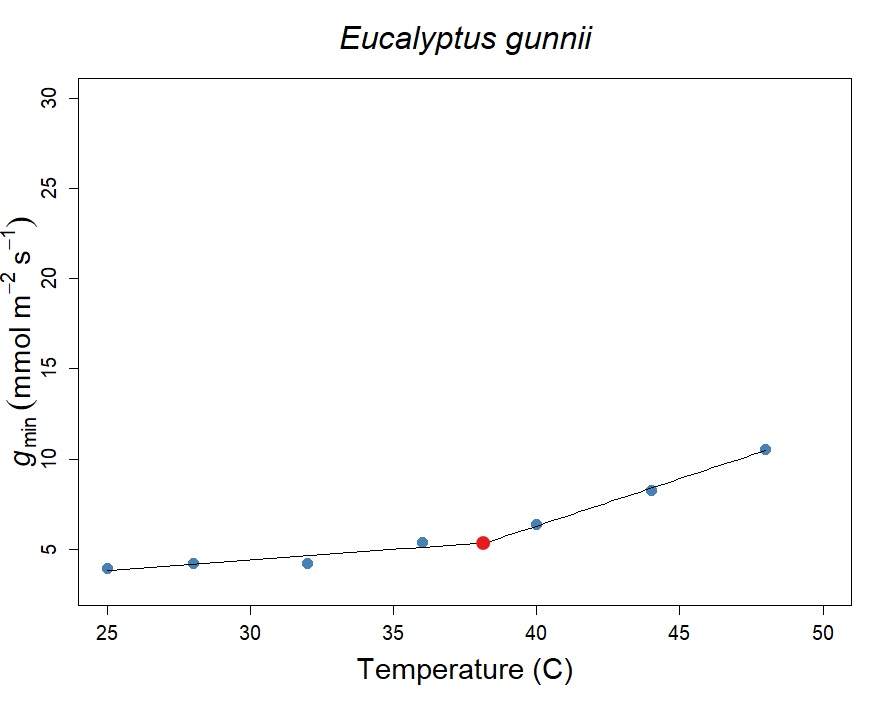


1. *E. johnstonii*


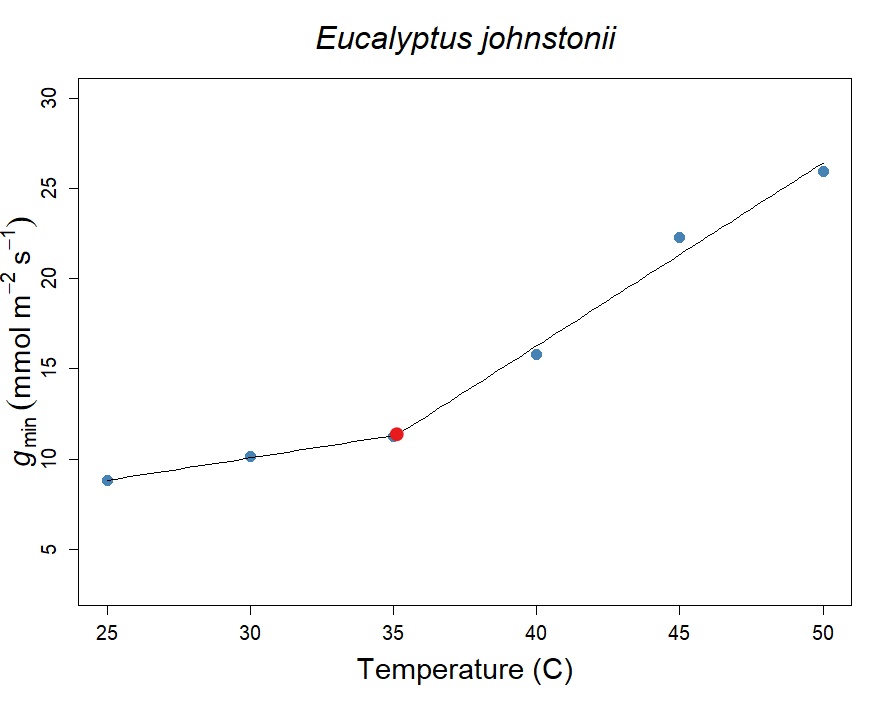


1. *E. vernicosa*


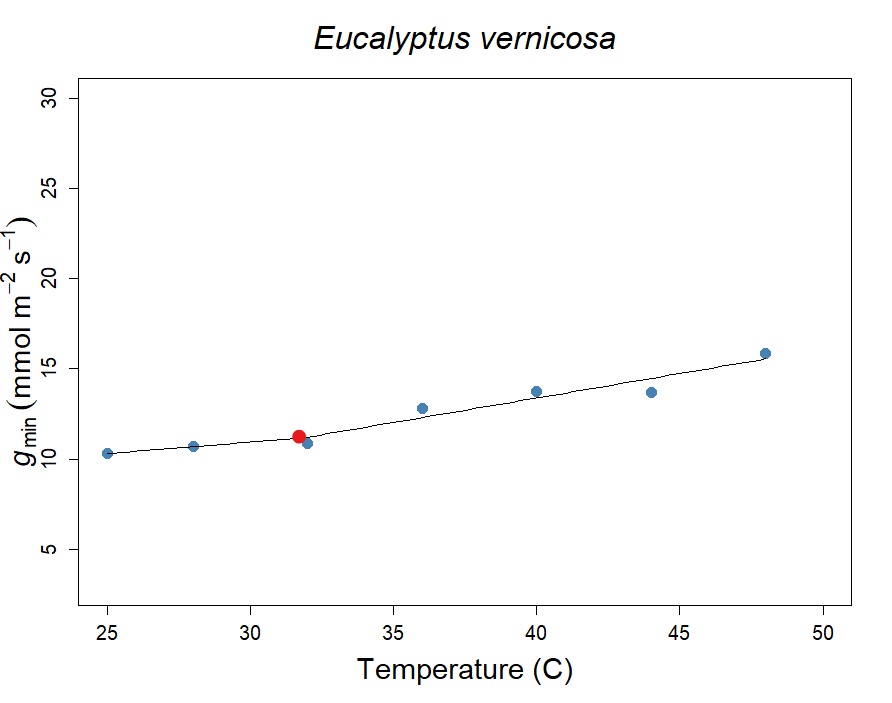


1. *E. viminalis*


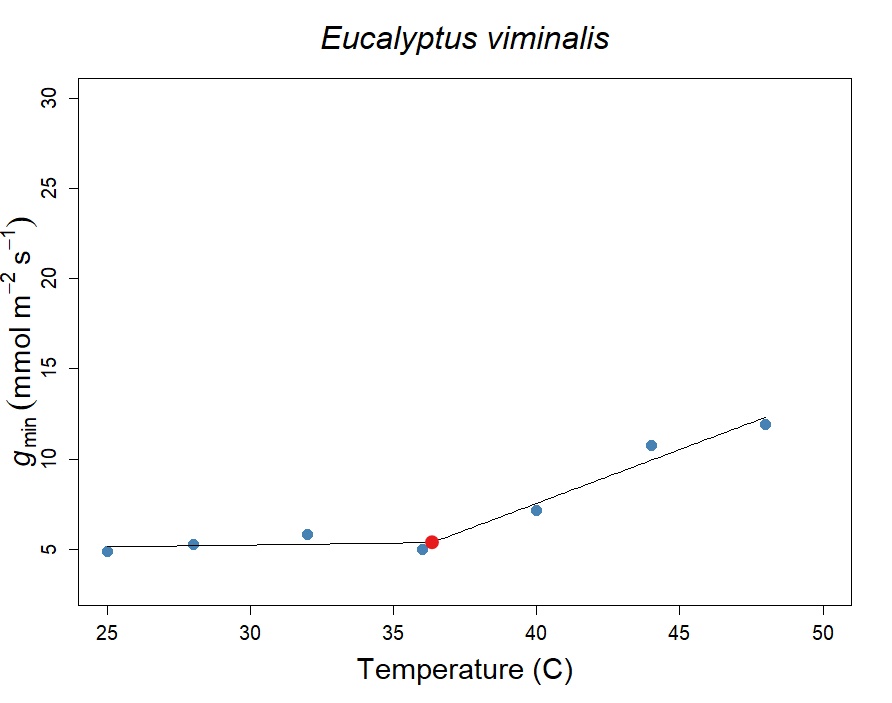


1. *E. dalrympleana*


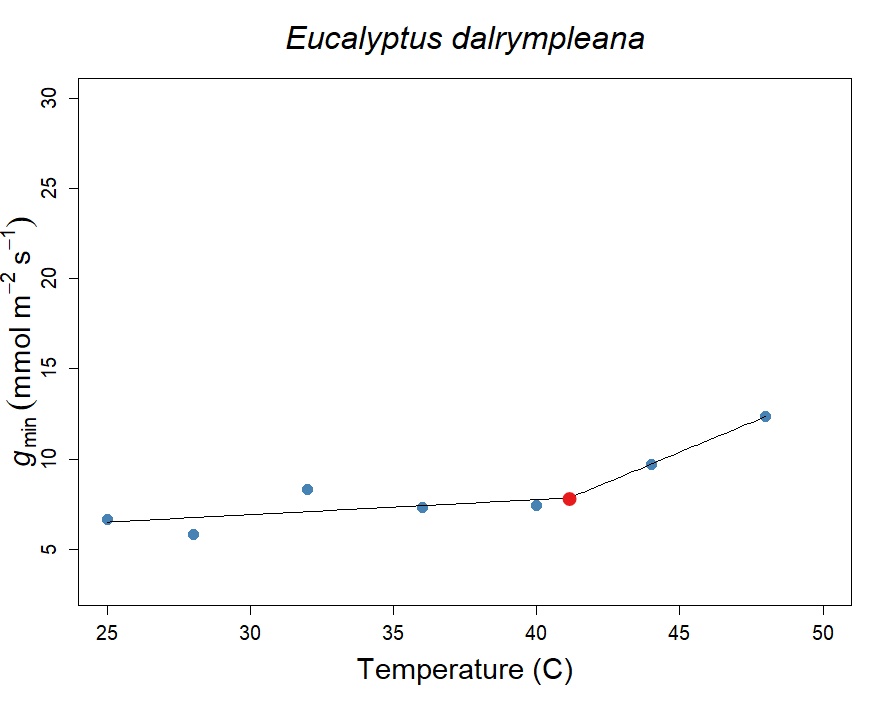


1. *E. amygdalina*


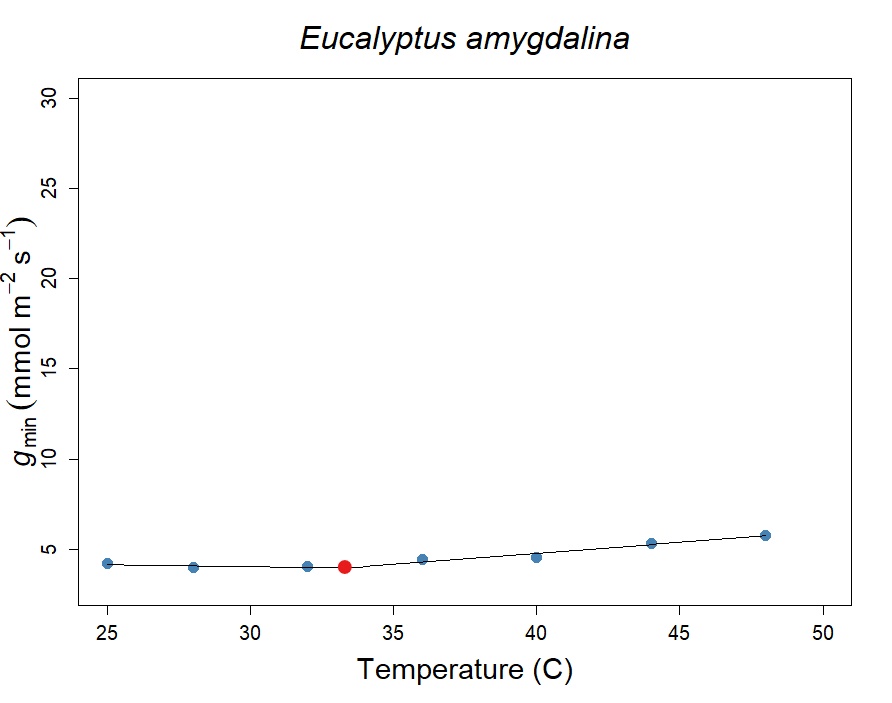


1. *E. coccifera*

*
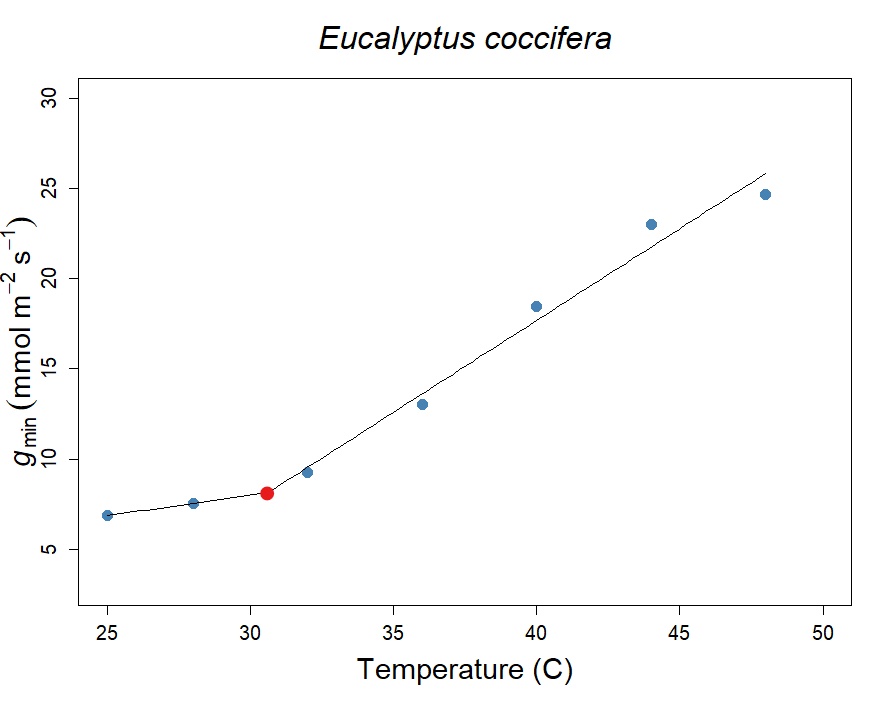
*

1. *E. nitida*


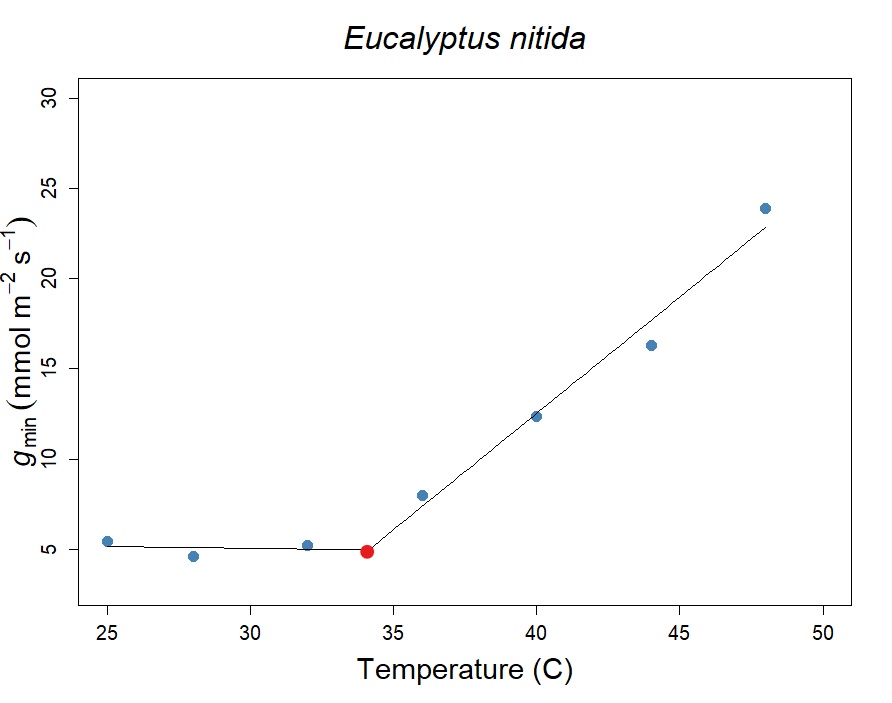


1. *E. pulchella*


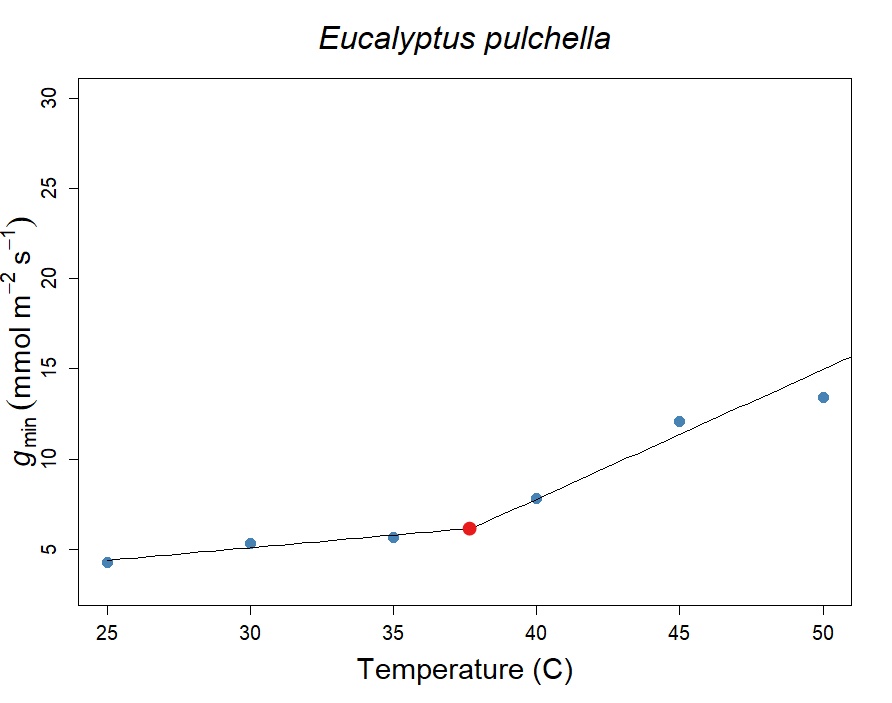


1. *E. regnans*


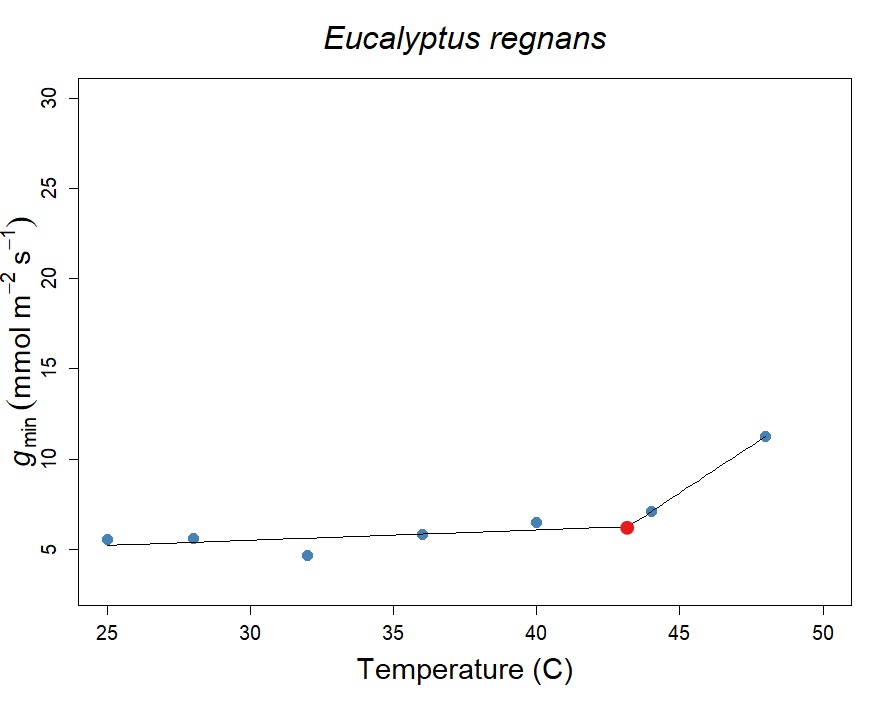


1. *E. risdonii*


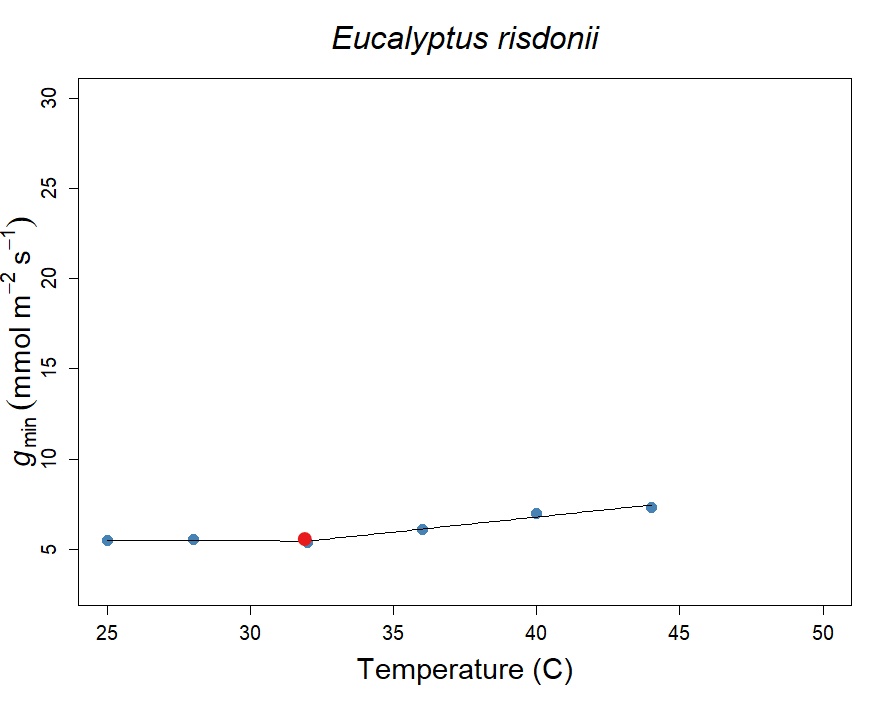


**Figure S2:** g_min_ (mmol m^-2^ s^-1^) plotted against temperature (˚C) as a mean of each of the five samples of fourteen Tasmanian *Eucalyptus* species: a) *E. archeri*, b) *E. barberi*, c) *E. globulus*, d) *E. gunnii*, e) *E. johnstonii*, f) *E. viminalis*, g) *E. vernicosa*, h) *E. dalrympleana*, i) *E. amygdalina*, j) *E. coccifera*, k) *E. nitida*, l) *E. pulchella*, m) *E. regnans*, and n) *E. risdonii*. g_min_, at the standard 25˚C, ranged from 3.04 ± 2.28 mmol m^-2^ s^-1^ (*E. risdonii*) to 10.01 ± 2.82 mmol m^-2^ s^-1^ (*E. vernicosa*). T_P_ (˚C) is represented by the breakpoint (indicated in red) of the regression line, denoting the two distinct phases (a phase of consistent water loss, followed by a phase of increasing water loss. T_P_ ranged from 31.85 ˚C (*E. vernicosa*) to 43.20 ˚C (*E. regnans*).

|  |  |  |  |
| --- | --- | --- | --- |


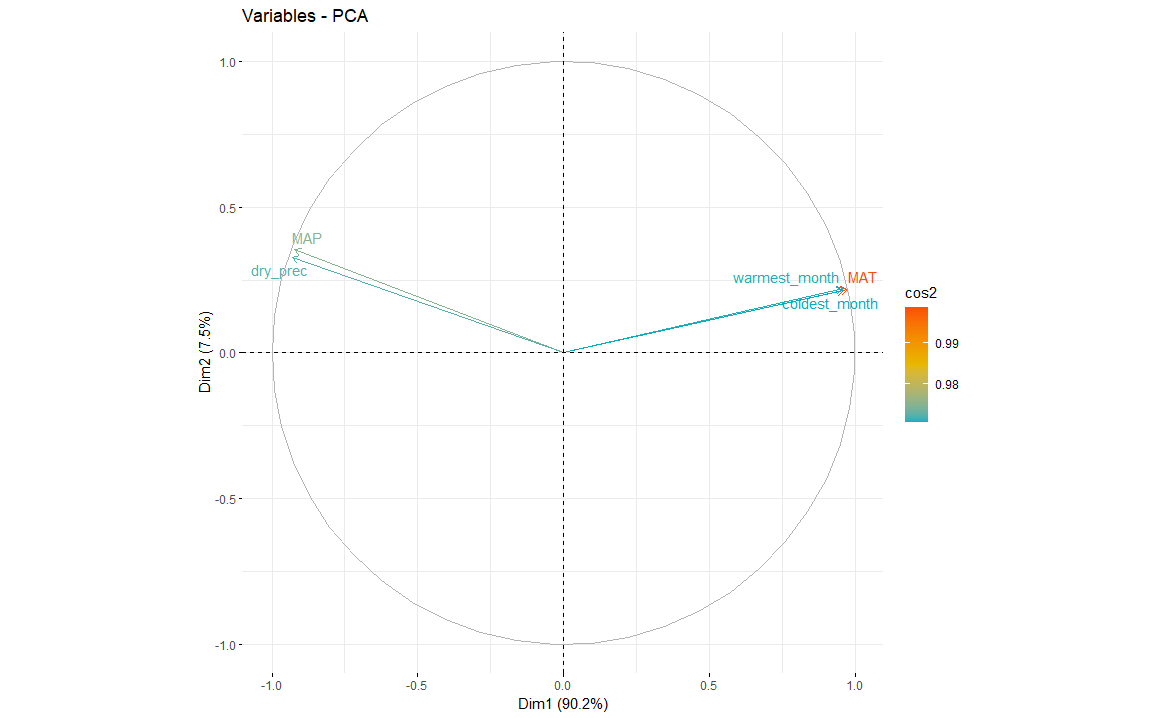


**Figure S3:** Principal component analysis (PCA) including five climate variables (mean annual temperature (MAT), mean annual precipitation (MAP), maximum temperature in the warmest month (warmest_month), minimum temperature in the coldest month (coldest_month), and precipitation in the driest quarter (dry_prec)). PC1 accounted for 90.2% of variance and PC2 accounted for 7.5%.


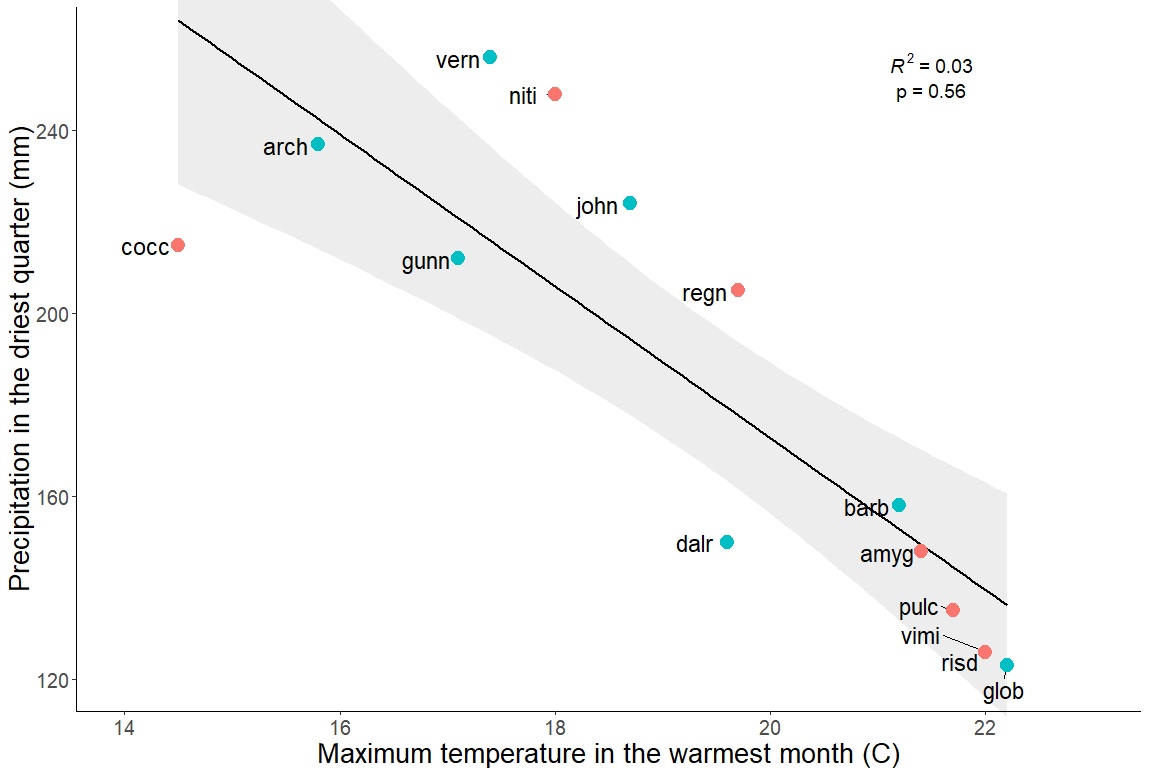


**Figure S4:** Correlation (including confidence interval) between maximum temperature in the warmest month (˚C) and precipitation in the driest quarter (mm) at sampling sites of fourteen Tasmanian *Eucalyptus* species. Species are labelled according to their four letter abbreviation (Table 1). Subgenus is represented by point colour (*Symphyomyrtus* in blue and *Eucalyptus* in red).


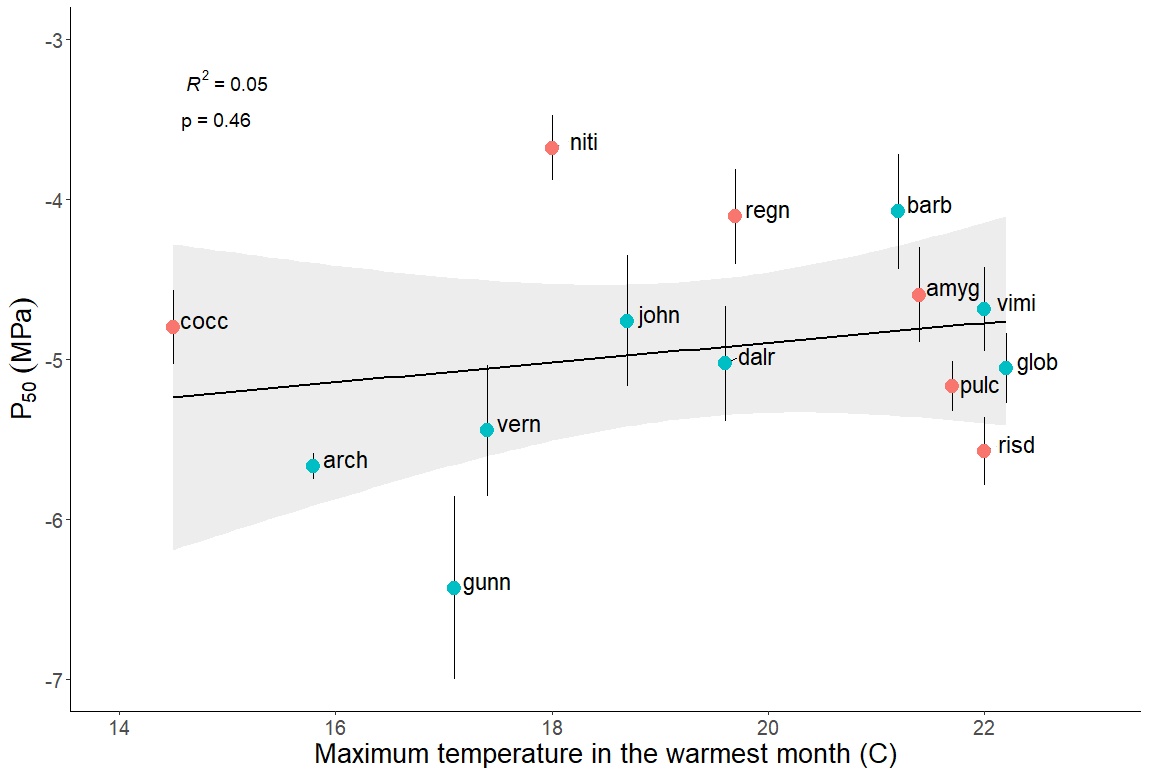


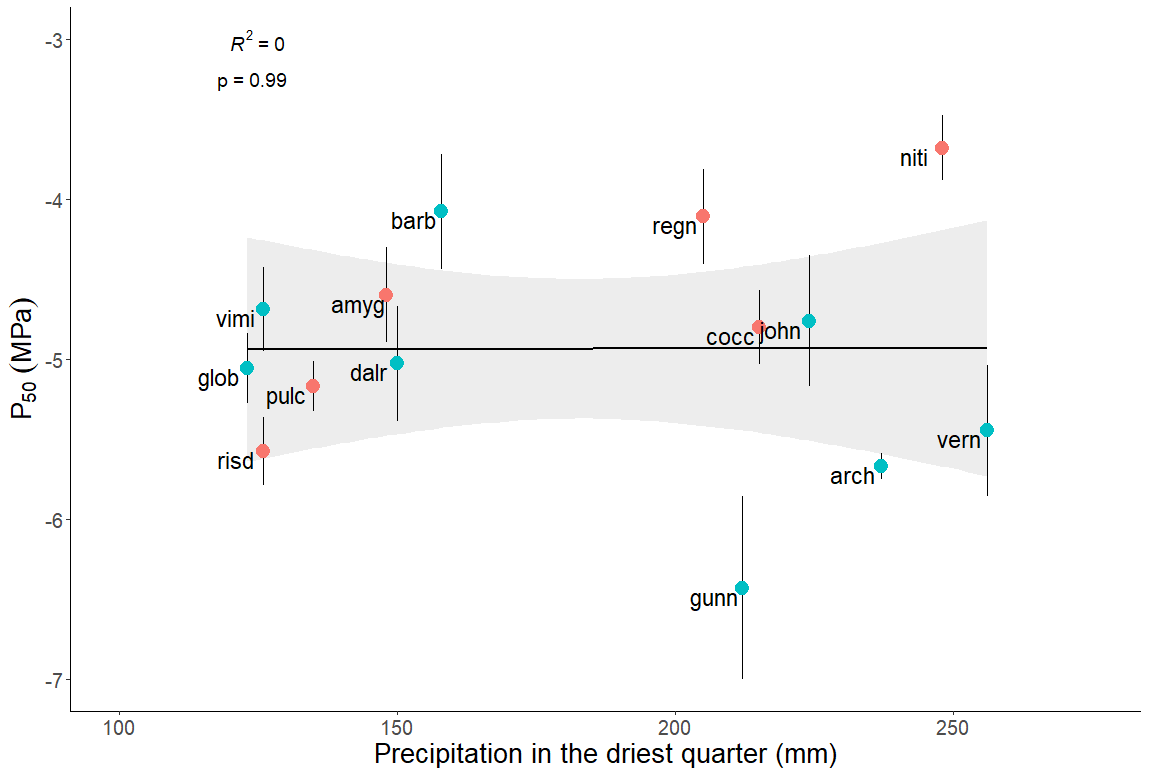


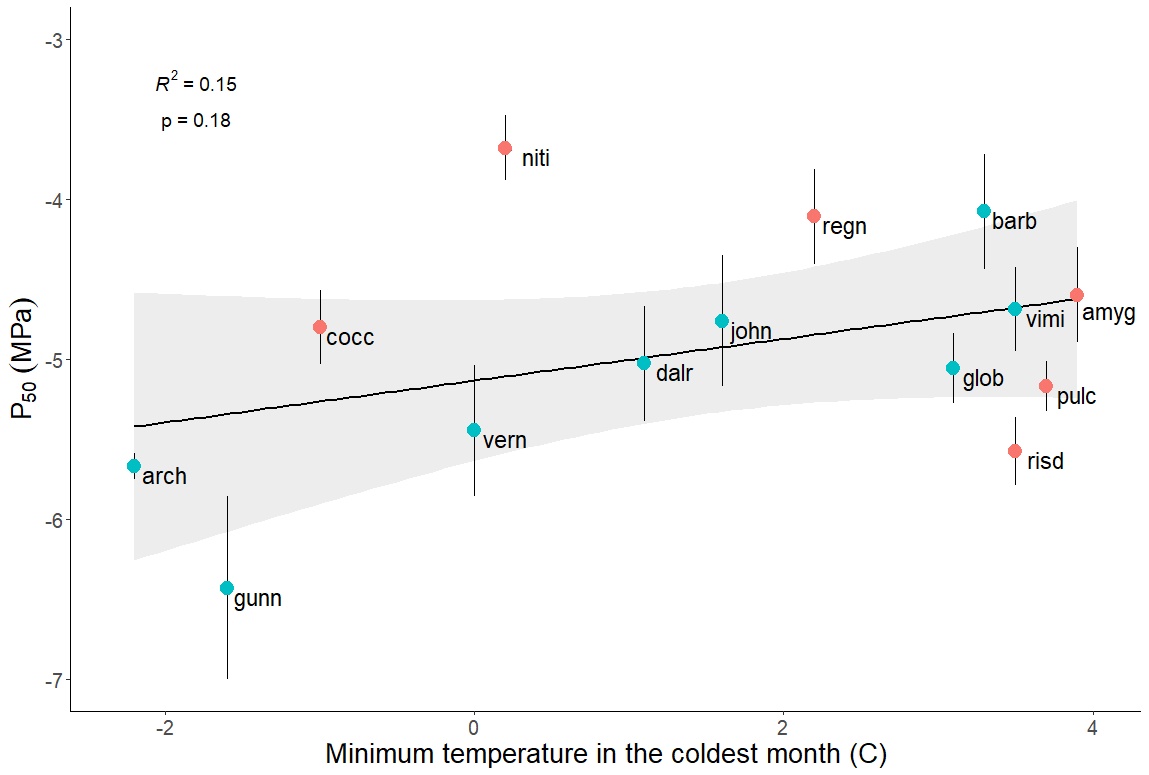


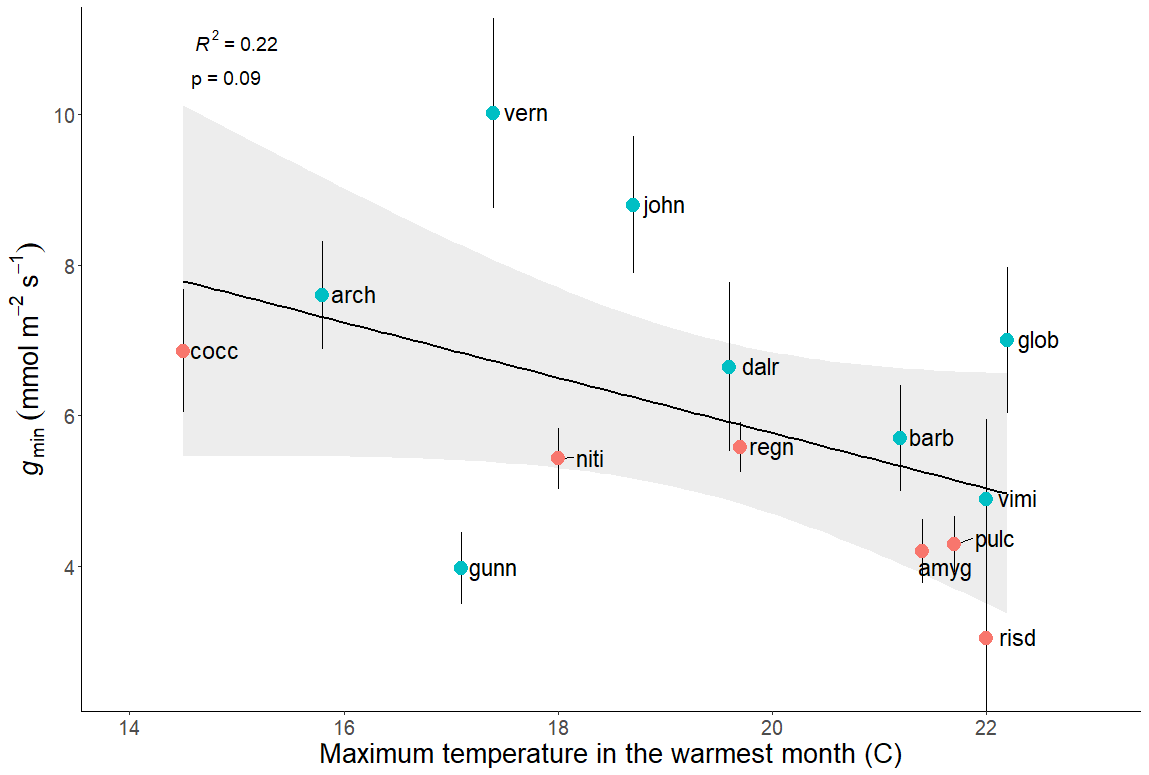


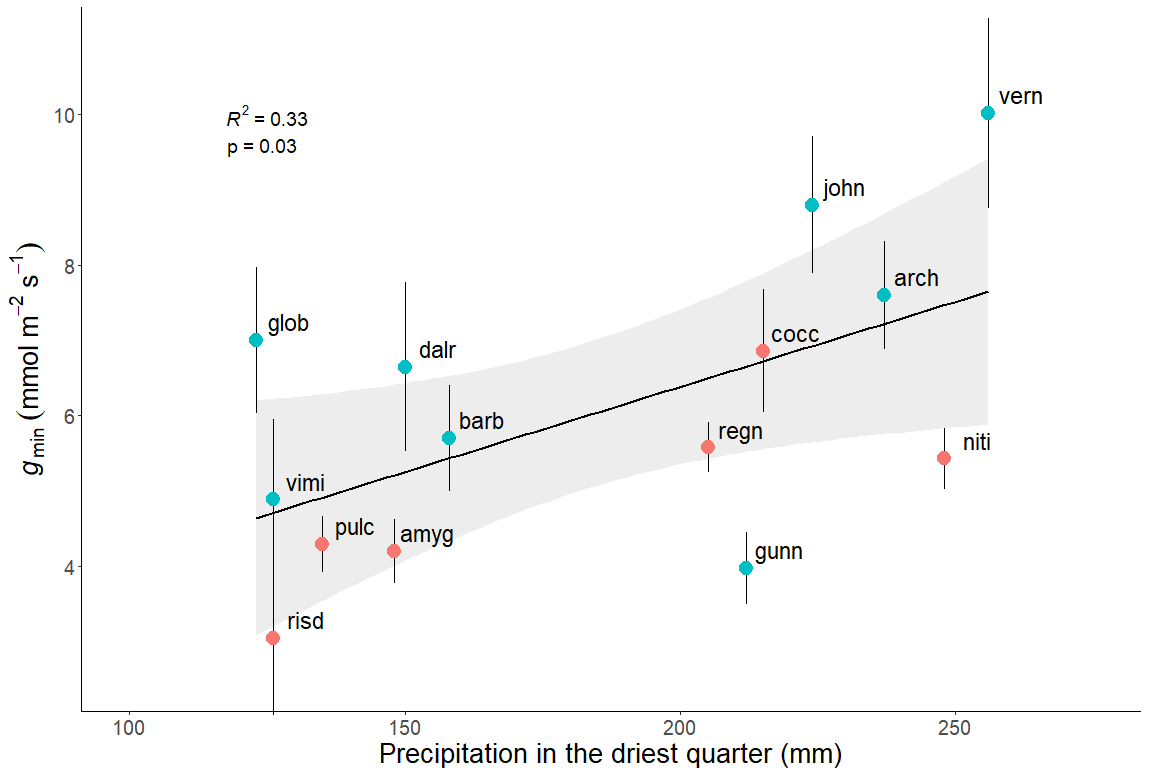


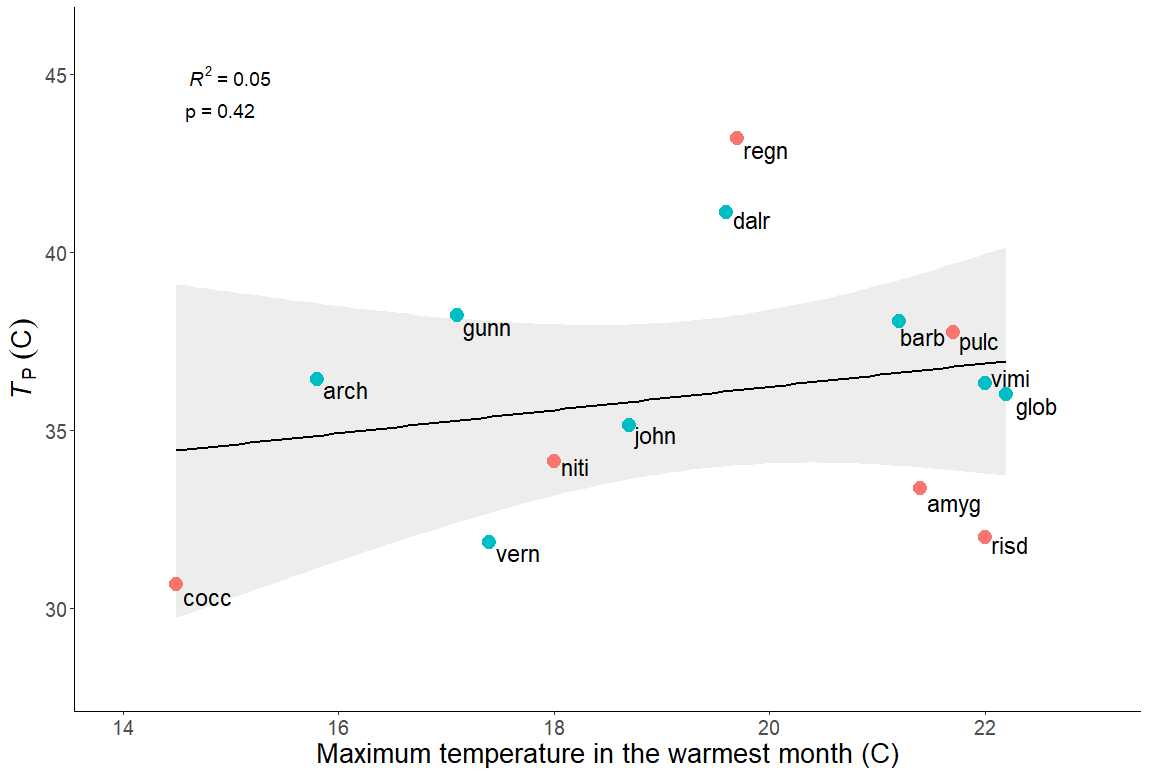


g)


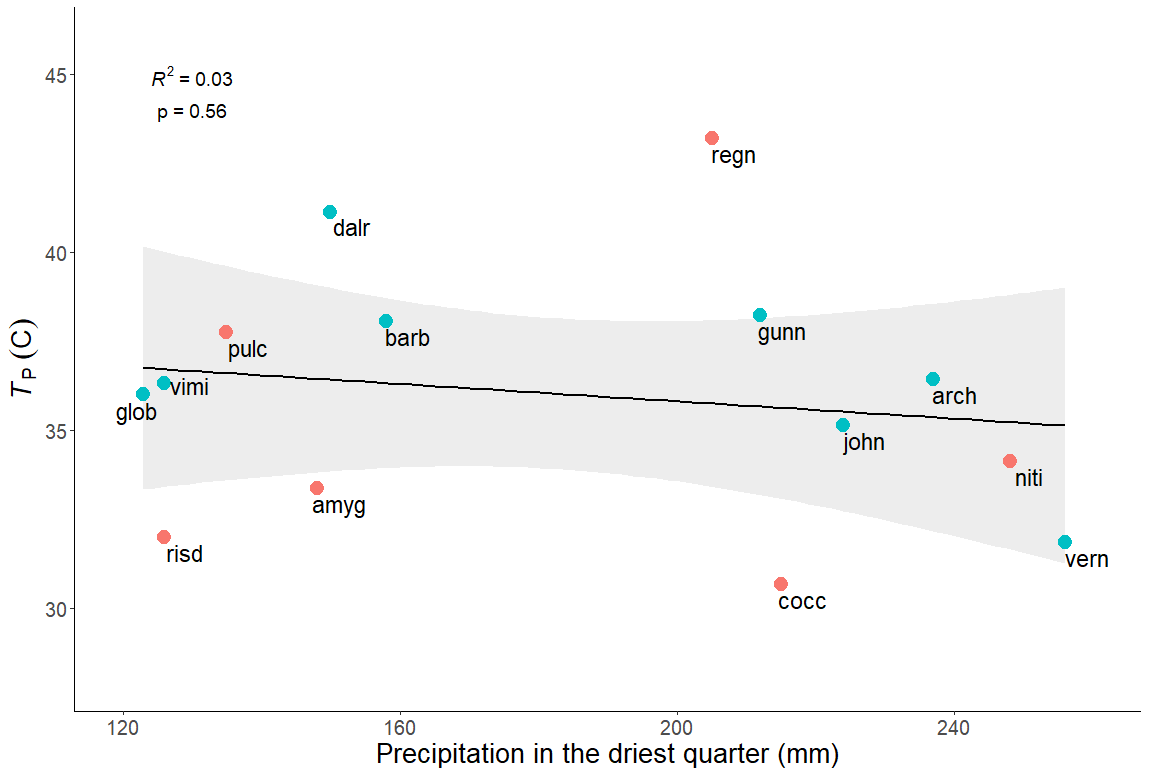


**Figure S5:** Phylogenetic generalised least squares (PGLS) regressions (including confidence intervals for regression lines and standard error bars for relevant traits) for physiological traits and individual climate variables. These climate variables include maximum temperature in the warmest month (C˚) and precipitation in the driest quarter (mm) (for P_50_ (a, b), *g*_min_ (d, e), and T_P_ (f, g)) and minimum temperature in the coldest month (C˚) (for P_50_; c). Species are labelled according to their four letter abbreviation (Table 1). Subgenus is represented by point colour (*Symphyomyrtus* in blue and *Eucalyptus* in red).

**
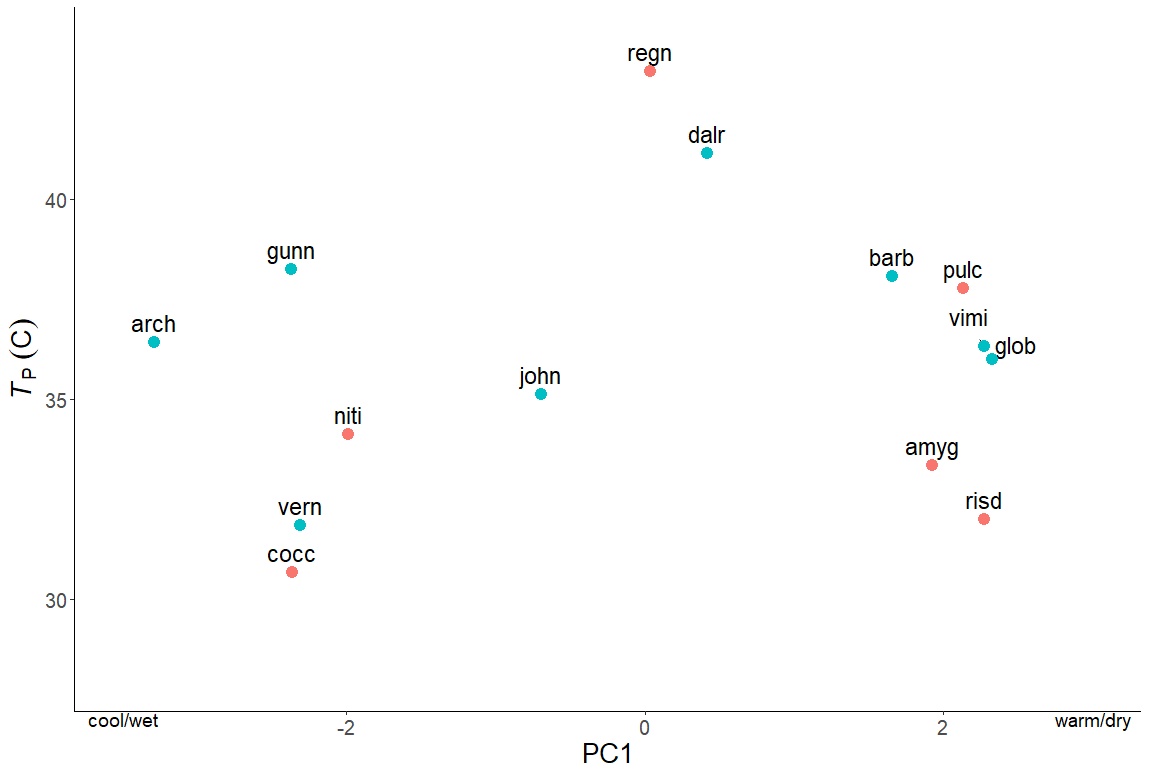
**

**Figure S6:**

Phylogenetic generalised least squares (PGLS) regression for PC1 (with cooler/wetter climates to the left and warmer/drier climates to the right) and *T*_P_ (R^2^ = -0.08, p = 0.62). Species are labelled according to their four letter abbreviation (Table 1). Subgenus is represented by point colour (*Symphyomyrtus* in blue and *Eucalyptus* in red).
