# Supplementary material for: Cold temperature and aridity shape the evolution of drought tolerance traits in Tasmanian species of Eucalyptus
Source: Tree Physiol. 2023 May 19;43(9):1493–500. doi: 10.1093/treephys/tpad065 (PMC10493950; doi:10.1093/treephys/tpad065)
Supplement: Supplementary_Tables_tpad065 [file supplementary_tables_tpad065.docx]

Supplementary Tables

**Table S1:**

Maximum temperature in the warmest month (˚C) and minimum aridity index (mean annual precipitation divided by mean annual potential evapotranspiration, based on a monthly average (Zomer et al. 2007)) across the Tasmanian range of each species chosen in this study. The site latitude, longitude, and elevation (m), population mean tree heights (m), and species mean specific leaf area (SLA; m^2^ kg^-1^) for each sampling site is also indicated.

| Species | Temperature (˚C) | Aridity | Sampling site | Latitude | Longitude | Elevation (m) | Tree height (m) | SLA (m^2^ kg^-1^) |
| --- | --- | --- | --- | --- | --- | --- | --- | --- |
| E. archeri | 20 | 0.82 | Projection Bluff | -41.72 | 146.72 | 1092 | 6 | 0.0040 |
| E. barberi | 21 | 0.50 | Cherry Tree Hill | -41.98 | 148.14 | 180 | 2 | 0.0037 |
| E. dalrympleana | 22 | 0.53 | Moogara | -42.80 | 146.92 | 355 | 20 | 0.0046 |
| E. globulus | 21 | 0.50 | Tea Tree | -42.68 | 147.40 | 90 | 20 | 0.0034 |
| E. gunnii | 17.76 | 0.78 | Pine Tree Rivulet | -41.80 | 146.68 | 1053 | 15 | 0.0041 |
| E. johnstonii | 19 | 0.75 | The Springs | -42.92 | 147.25 | 694 | 10 | 0.0034 |
| E. vernicosa | 18.02 | 1.46 | Tim Shea | -42.72 | 146.46 | 877 | 0.5 | 0.0028 |
| E. viminalis | 22 | 0.41 | Risdon Hills | -42.83 | 147.33 | 110 | 7 | 0.0047 |
| E. amygdalina | 22 | 0.45 | Peter Murrell | -43.00 | 147.30 | 63 | 12 | 0.0050 |
| E. coccifera | 20 | 0.72 | Big Bend | -42.89 | 147.22 | 1102 | 5 | 0.0037 |
| E. nitida | 21 | 0.58 | Tim Shea | -42.71 | 146.47 | 654 | 4 | 0.0039 |
| E. pulchella | 22 | 0.48 | Sandy Bay | -42.91 | 147.32 | 70 | 15 | 0.0049 |
| E. regnans | 25.68 | 0.51 | Neika | -42.93 | 147.25 | 432 | 30 | 0.0053 |
| E. risdonii | 22 | 0.48 | Risdon Hills | -42.83 | 147.33 | 110 | 5 | 0.0051 |

**Table S2:**

Phylogenetic signal using two methods (Blomberg’s K and Pagel’s lambda) for the three physiological traits (P_50 leaf_, *g*_min_, and *T*_P_) and the five climate variables (mean annual temperature, mean annual precipitation, precipitation in the driest quarter, maximum temperature in the warmest month, and minimum temperature in the coldest month). Signal strength and p-value are reported for each variable and method.

| Trait/Climate Variable | Method | Signal | p-value |
| --- | --- | --- | --- |
| P_50 leaf_ | lambda | 0.00 | 1.00 |
| P_50 leaf_ | K | 0.01 | 0.39 |
| g_min_ | lambda | 0.14 | 0.47 |
| g_min_ | K | 0.02 | 0.04 |
| T_P_ | lambda | 0.00 | 1.00 |
| T_P_ | K | 0.01 | 0.43 |
| Mean annual temperature | lambda | 0.00 | 1.00 |
| Mean annual temperature | K | 0.02 | 0.29 |
| Mean annual precipitation | lambda | 0.00 | 1.00 |
| Mean annual precipitation | K | 0.02 | 0.26 |
| Precipitation in the driest quarter | lambda | 0.00 | 1.00 |
| Precipitation in the driest quarter | K | 0.02 | 0.22 |
| Maximum temperature in the warmest month | lambda | 0.00 | 1.00 |
| Maximum temperature in the warmest month | K | 0.01 | 0.35 |
| Minimum temperature in the coldest month | lambda | 0.00 | 1.00 |
| Minimum temperature in the coldest month | K | 0.02 | 0.24 |
